# Type II collagen-positive progenitors are important stem cells in controlling skeletal development and vascular formation

**DOI:** 10.1038/s41413-022-00214-z

**Published:** 2022-06-23

**Authors:** Xinhua Li, Shuting Yang, Gongsheng Yuan, Dian Jing, Ling Qin, Hu Zhao, Shuying Yang

**Affiliations:** 1 Department of Basic and Translational Sciences, School of Dental Medicine, University of Pennsylvania, Philadelphia, PA 19104 USA; 2 Department of Orthopedics, Shanghai General Hospital, Shanghai Jiao Tong University, School of Medicine, Shanghai, 200080 P. R. China; 3 Department of Spinal Surgery, East Hospital, Tongji University, School of Medicine, Shanghai, 200120 China; 4 Department of Restorative Sciences, College of Dentistry, Texas A&M University, Dallas, TX USA; 5 Department of Orthopedic Surgery, Perelman School of Medicine, University of Pennsylvania, Philadelphia, PA 19104 USA; 6 The Penn Center for Musculoskeletal Disorders, School of Medicine, University of Pennsylvania, Philadelphia, PA 19104 USA; 7 Center for Innovation & Precision Dentistry, School of Dental Medicine, School of Engineering and Applied Sciences, University of Pennsylvania, Philadelphia, PA 19104 USA

**Keywords:** Bone, Pathogenesis

## Abstract

Type II collagen-positive (Col2^+^) cells have been reported as skeletal stem cells (SSCs), but the contribution of Col2^+^ progenitors to skeletal development both prenatally and postnatally during aging remains unclear. To address this question, we generated new mouse models with ablation of Col2^+^ cells at either the embryonic or postnatal stages. The embryonic ablation of Col2^+^ progenitors resulted in the death of newborn mice due to a decrease in skeletal blood vessels, loss of all vertebral bones and absence of most other bones except part of the craniofacial bone, the clavicle bone and a small piece of the long bone and ribs, which suggested that intramembranous ossification is involved in long bone development but does not participate in spine development. The postnatal ablation of Col2^+^ cells resulted in mouse growth retardation and a collagenopathy phenotype. Lineage tracing experiments with embryonic or postnatal mice revealed that Col2^+^ progenitors occurred predominantly in the growth plate (GP) and articular cartilage, but a limited number of Col2^+^ cells were detected in the bone marrow. Moreover, the number and differentiation ability of Col2^+^ progenitors in the long bone and knee joints decreased with increasing age. The fate-mapping study further revealed Col2^+^ lineage cells contributed to, in addition to osteoblasts and chondrocytes, CD31^+^ blood vessels in both the calvarial bone and long bone. Specifically, almost all blood vessels in calvarial bone and 25.4% of blood vessels in long bone were Col2^+^ lineage cells. However, during fracture healing, 95.5% of CD31^+^ blood vessels in long bone were Col2^+^ lineage cells. In vitro studies further confirmed that Col2^+^ progenitors from calvarial bone and GP could form CD31^+^ vascular lumens. Thus, this study provides the first demonstration that intramembranous ossification is involved in long bone and rib development but not spine development. Col2^+^ progenitors contribute to CD31^+^ skeletal blood vessel formation, but the percentage differs between long bone and skull bone. The number and differentiation ability of Col2^+^ progenitors decreases with increasing age.

## Introduction

Endochondral and intramembranous ossification are two processes involved in bone development in both humans and mice. The main difference between endochondral and intramembranous ossification is that the former forms bone through a cartilage intermediate, whereas the latter forms bone directly from neural crest-derived mesenchymal cells. Studies have shown that most bones in the body are formed through endochondral ossification, and only the bones of the cranial base and caudal cranial vault in the skull and clavicles ossify intramembranously.^1,2^

Cartilaginous matrix proteins such as type II collagen alpha 1 (Col2α1), aggrecan (Acan) and Sox9 are markers of chondrocytes. Mutation of Col2α1,^3–6^ Acan^7^ or Sox9^8,9^ causes severe skeletal malformation, including lower body weight, a deformed skeletal structure, severe growth retardation, malformation of endochondral bones and intervertebral discs, cleft palate and abnormal cartilage histomorphology. However, the contribution of type II collagen alpha 1-positive (Col2^+^) cells to skeletal development both prenatally and postnatally remains unclear.

Skeletal stem cells (SSCs) are defined by their multidifferentiation potential, such as their ability to differentiate into osteogenic, chondrogenic, and adipogenic progenies.^10,11^ Using lineage tracing techniques^12^ to trace the fate of leptin receptor-positive (Lepr^+^) cells,^13,14^ chemokine (C-X-C motif) ligand 12-positive (Cxcl12^+^) cells,^15^ Gli1^+^ cells,^16^ Prx^+^ cells,^17^ Nestin^+^ cells,^18^ Sox9^+^ cells,^19^ Acan^+^ cells^19^ and Ctsk^+^ cells,^20^ recent studies have revealed that these cells are all SSCs and play important roles during bone development and homeostasis. Importantly, Col2^+^ cells have been found to contribute to chondrocytes, osteoblasts, early perichondrial precursors before Runx2 expression, Cxcl12-abundant stromal cells and bone marrow stromal/mesenchymal progenitor cells during postnatal life. Furthermore, Col2α1 is expressed by progenitors of the skeletal lineage during canonical endochondral bone formation in the cranial base.^19,21^ However, how Col2^+^ progenitors contribute to skeletal development during aging remains unclear.

Here, by generating mice with ablation of Col2^+^ cells using a diphtheria toxin (DTA) transgenic line and genetic lineage tracing mouse models with tdTomato transgenic lines, we determined the function of Col2^+^ cells in the embryonic and postnatal stages. Our findings provide the first demonstration that Col2^+^ cells contribute to calvarial bone development, that intramembranous ossification derived from Col2-negative progenitors is involved in long bone and rib development but does not participate in spine development, and that Col2^+^ cells also contribute to blood vessel formation during skeletal development and fracture healing.

## Results

### The ablation of Col2^+^ cells causes the death of newborn mice with loss of all vertebral bone and most other bones and cartilage

To understand the contribution of Col2^+^ progenitors to skeletal development, a new mouse model of Col2-cre;DTA^+/−^ was created by breeding mice bearing a DTA transgene downstream of a floxed stop codon (DTA^+/+^) with Col2-cre mice. Cre- littermates served as controls (wild-type mice, WT mice) (Fig. S1A). On embryonic day 17.5 (E17.5), the Col2-cre;DTA^+/−^ embryos survived and followed Mendelian law (19/80). Interestingly, we found that 70%–75% of mice died between E17.5 and the newborn stage. Approximately 25% of the newborn (16/72) Col2-cre;DTA^+/−^ mice survived for minutes or a few hours after birth and then died due to oxygen insufficiency resulting from a lack of rib cages and other developmental defects.

**Fig. 1:**
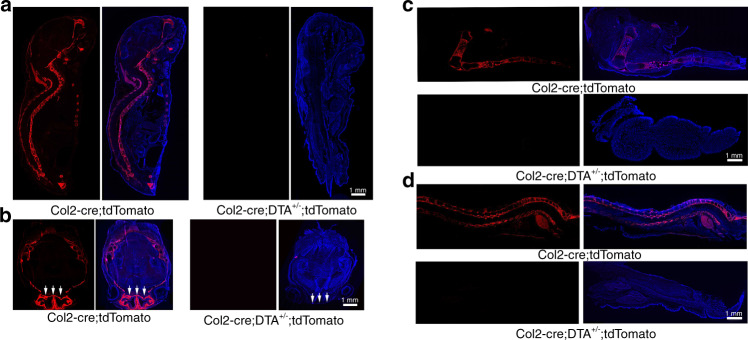
Spatial expression of Col2^+^ lineage progenitors in newborns and removal of the majority of Col2^+^ cells by DTA. **a** Fluorescence images showing the pattern of Col2^+^ lineage progenitors in middle sections of Col2-cre;tdTomato and Col2-cre;DTA^+/−^;tdTomato newborn mice. **b** Fluorescence images showing the pattern of Col2^+^ lineage progenitors in transected skulls of Col2-cre;tdTomato and Col2-cre;DTA^+/−^;tdTomato mice. White arrow, defective nasal capsule in a Col2-cre;DTA^+/−^;tdTomato mouse. **c** Fluorescence images showing the pattern of Col2^+^ lineage progenitors in the lower extremities of Col2-cre;tdTomato and Col2-cre;DTA^+/−^;tdTomato newborn mice. Note the complete loss of tdTomato^+^ cells in the long bones of Col2-cre;DTA^+/−^;tdTomato mice compared with wild-type newborn mice. **d** Fluorescence images showing the pattern of Col2^+^ lineage progenitors in middle sagittal sections of spines of Col2-cre;tdTomato and Col2-cre;DTA^+/−^;tdTomato newborn mice. *n* = 6 mice per condition; three independent experiments

To test the effect of Cre-mediated DTA ablation, we generated Col2-cre;tdTomato and Col2-cre;DTA^+/−^;tdTomato mice. Lineage tracing detected abundant levels of tdTomato^+^ cells in the spine, limbs, ribs, meninges and skull bone of Col2-cre;tdTomato mice (Fig. 1a), but few tdTomato^+^ cells were detected in the mutant mice compared with the WT mice. Notably, we found that many cells in the WT skull bone, particularly in the cartilaginous nasal capsule, were tdTomato^+^ (Fig. 1b), which confirmed that Col2^+^ cells contribute to skull bone and nasal capsule formation. Consistently, the cartilaginous nasal capsule was completely lost in the Col2-cre;DTA^+/−^;tdTomato newborns, and the skulls of these newborns were smaller ^+/−^ than those of the control newborns. Most cells in the lower extremities and spine were labeled, although articular chondrocytes exhibited a weak signal (Fig. 1c, d). In contrast, no tdTomato^+^ cells could be detected in the spine region and lower extremities of Col2-cre;DTA^+/−^;tdTomato newborns, which confirmed the effectiveness of cell ablation.

**Fig. 2:**
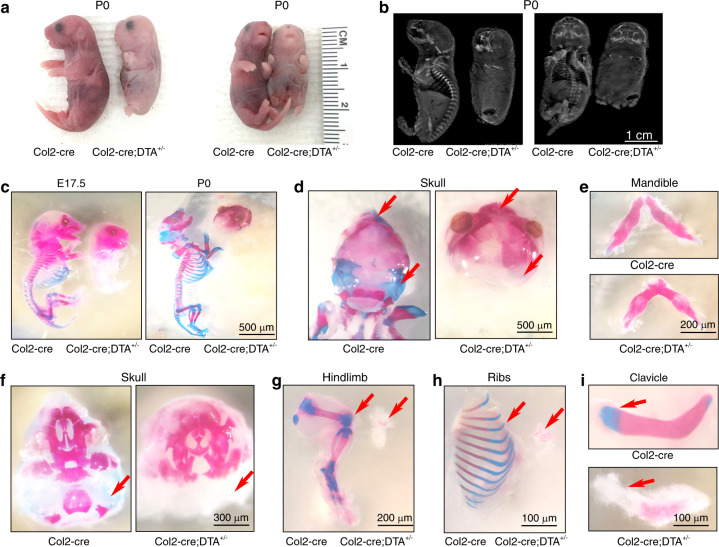
Ablation of Col2^+^ cells causes lethality in newborn mice due to the absence of endochondral bone and cartilage. **a** Gross appearance of Col2-cre and Col2-cre;DTA^+/−^ mice at P0. The mutant mouse is small and has an extremely short four-limb pattern. **b** X-ray of P0 wild-type and mutant embryos. **c** Skeletons of Col2-cre and Col2-cre;DTA^+/−^ mice at E17.5 and the newborn stage. Embryos and newborns were double stained with Alizarin red/Alcian blue. **d** Alizarin red/Alcian blue staining of skulls of Col2-cre and Col2-cre;DTA^+/−^ newborns. Red arrow, complete loss of cartilage in Col2-cre;DTA^+/−^ mice. **e** Alizarin red/Alcian blue staining of the mandibles of Col2-cre and Col2-cre;DTA^+/−^ newborn mice. **f** Interior view showing the lack of mandibles in the skulls of Col2-cre and Col2-cre;DTA^+/−^ newborn mice. Red arrow, bone loss in Col2-cre;DTA^+/−^ mice. **g** Hindlimbs of Col2-cre and Col2-cre;DTA^+/−^ newborn mice. Red arrow, bone loss in Col2-cre;DTA^+/−^ mice. **h** Ribs of Col2-cre and Col2-cre;DTA^+/−^ newborn mice. Red arrow, bone loss in Col2-cre;DTA^+/−^ mice. **i** Clavicles of Col2-cre and Col2-cre;DTA^+/−^ newborn mice. Red arrow, partial clavicle loss in Col2-cre;DTA^+/−^ mice. *n* = 6 mice per condition; three independent experiments

The gross appearance of the Col2-cre;DTA^+/−^ newborn mice showed severe dwarfism with extremely shortened limbs, tail and nose, intact but pale skin, cleft palate, and abnormal skull morphology^22^ (Fig. 2a, Fig. S1B, C).

A skeletal radiograph examination of Col2-cre;DTA^+/−^ newborns revealed that their vertebral bone was completely lost, and only parts of the skull, mandible, clavicle bone, and tiny bones of the hindlimbs and ribs were observed (Fig. 2b). Consistent with the X-ray results, Alizarin red/Alcian blue staining of mutant embryos at E17.5 revealed the absence of cartilage and bone throughout the body skeleton and only limited Alizarin red staining in the craniofacial bone (Fig. 2c), including the frontal, parietal, temporal, maxillary, interparietal and supraoccipital bones in the skull (Fig. 2d, f) and mandible (Fig. 2e). Interestingly, very small pieces of bone in the hindlimb (Fig. 2G), small parts of the ribs (Fig. 2h), and the clavicle bone were calcified with positive Alizarin red staining (Fig. 2i).

### Ablation of Col2^+^ cells disrupted bone and cartilage development

**Fig. 3:**
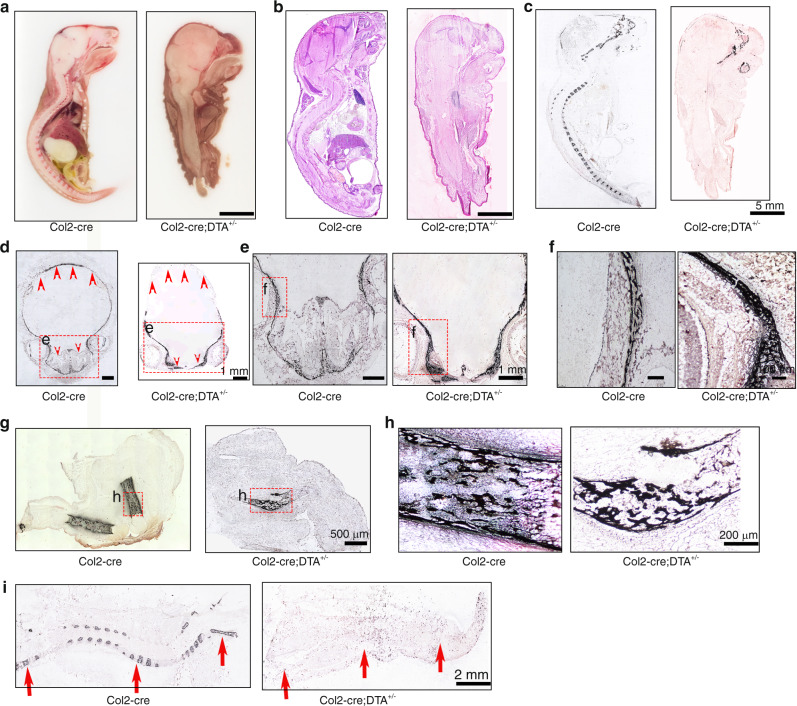
Ablation of Col2^+^ cells results in absence of the spine and disruption of endochondral skeletal development. **a** Total view of middle sagittal sections of Col2-cre and Col2-cre;DTA^+/−^ mice at P0. **b** H&E staining of the middle sagittal sections of Col2-cre and Col2-cre;DTA^+/−^ mice at P0. **c** Von Kossa staining of the middle sagittal sections of Col2-cre and Col2-cre;DTA^+/−^ mice at P0. **d** Von Kossa staining of the skull with transection. Yellow arrow, severe bone loss in the back of the skull of Col2-cre;DTA^+/−^ mice. Yellow star, nasal cavity area in Col2-cre and Col2-cre;DTA^+/−^ mice. **e** Von Kossa staining of craniofacial bone showing loss of the nasal cavity in mutant mice. **f** Higher magnification of Von Kossa staining showing similar sponge-like bone structures in both wild-type and mutant mice. **g** Von Kossa staining of bone in the hindlimbs of wild-type and mutant mice. **h** Higher magnification of Von Kossa staining of the hindlimbs of wild-type and mutant mice. Note that a small piece of well-calcified sponge-like bone structure can be detected in mice with ablation of Col2^+^ cells. **i** Von Kossa staining of the middle sagittal section of the spine of wild-type and mutant mice. Red arrow, severe vertebral bone loss in Col2-cre;DTA^+/−^ mice. *n* = 6 mice per condition; three independent experiments

To shed more light on the function of Col2^+^ progenitors in mice, we performed sagittal sectioning of Col2-cre control and Col2-cre;DTA^+/−^ newborns. In the Col2-cre newborns, all the bone, cartilage, brain, spinal cord, and organs were well organized and clearly identified (Fig. 3a). However, the entire vertebrae and intervertebral disc, most of the long bone and other bones and all the cartilage were lost in the Col2-cre;DTA^+/−^ mice (Fig. 3a). Most interestingly, despite the severe bone loss in the skull and complete absence of the vertebrae and intervertebral discs observed in the mutant mice, their brain and spinal cord were located in a relatively narrow skull cavity and a “spinal canal”, respectively. However, the morphology of the spinal cord presented a spindle-like structure, which suggested that the spine or vertebral bone may be needed to guide the formation of normal spinal cord morphology.

To gain further insights into the bone changes, histological sections of the skeletons from newborns were examined by H&E (Fig. 3b, Fig. S1D–F) and Von Kossa staining (Fig. 3c). Consistently, we found that the ablation of Col2^+^ cells resulted in the absence of most parts of the skeleton, including complete loss of the vertebrae and intervertebral disc and all body bones with the exception of parts of the skull and a tiny hindlimb bone, in the Col2-cre;DTA^+/−^ mice. To determine the defects in finer detail, we further analyzed sections of the skull, hindlimbs and spine. The results showed that only the front skull bone could be detected in the Col2-cre;DTA^+/−^ mice, and the nasal cavity and back skull bone were almost absent in these mice compared with the Cre control mice (Fig. 3d–f), which indicated that Col2^+^ cells also play an important role in skull development. Most interestingly, a small piece of calcified sponge-like bone structure could be detected in the hindlimbs of the Col2^+^ cell-ablated mice (Fig. 3g, h), which indicated that Col2^+^ cells dominantly contribute to long bone development but that Col2-negative (Col2^−^) cells also participate in long bone development. The Von Kossa staining results showed that vertebral bone mineralization was completely absent in Col2-cre;DTA^+/−^ mice, which suggested that Col2^+^ cells are the major progenitors contributing to vertebral bone development (Fig. 3i). Unexpectedly, even though almost all bones and cartilage were lost in Col2-cre;DTA^+/−^ newborns, the toe and finger digits and intact patterns were normal in these mice, which suggested that limb pattern development is independent of Col2^+^ cells and bone development in mice (Fig. S1B).

### The postnatal deletion of Col2^+^ cells resulted in mouse growth retardation and a type II collagenopathy phenotype

**Fig. 4:**
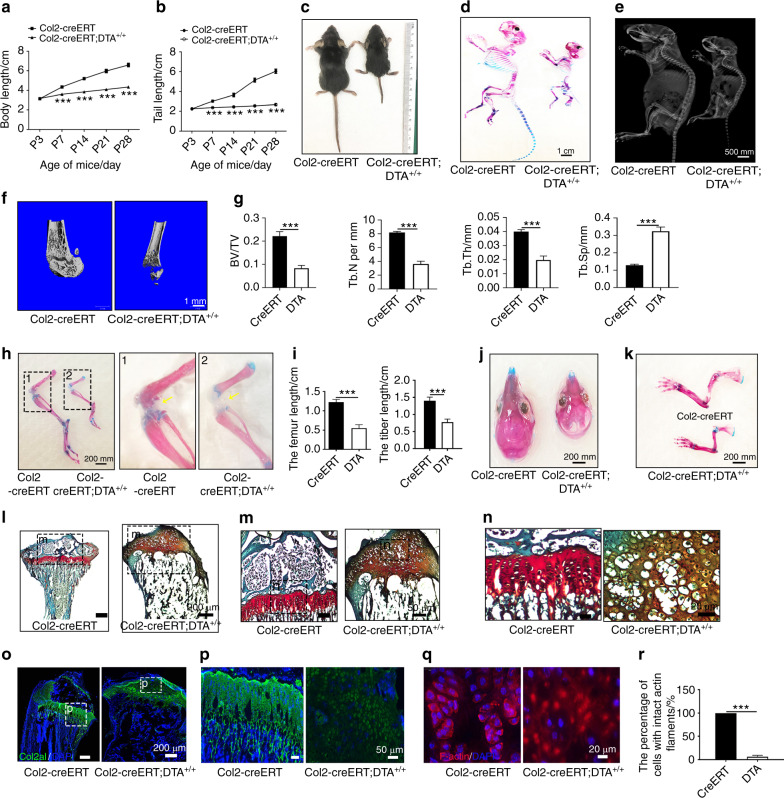
Postnatal deletion of Col2^+^ cells causes mouse growth retardation and a type II collagenopathy phenotype. **a**, **b** Quantitative analysis of the body length and tail length of Col2-creERT and Col2-creERT;DTA^+/+^ mice from P3 to four weeks of age (*n* = 6 mice per condition; three independent experiments). The data are presented as the means ± s.ds. **c** Macroscopic image of 4-week-old littermates. The mutant (right) mouse is small and exhibits dwarfism. **d** Total view of Col2-creERT and Col2-creERT;DTA^+/+^ mice double stained with Alizarin red and Alcian blue at four weeks of age. **e** X-ray of 4-week-old Col2-creERT and Col2-creERT;DTA^+/+^ mice. **f** Representative picture of the microCT 3D structure of the femur head of 4-week-old Col2-creERT and Col2-creERT;DTA^+/+^ mice. **g** Quantitative analysis of the BV/TV, Both, Tb.N, and Tb.Sp of the femurs of 4-week-old Col2-creERT (CreERT) and Col2-creERT;DTA^+/+^ (DTA) mice (*n* = 5 mice per group). The data are presented as the means ± s.ds. **h** Total view of the lower extremities of Col2-creERT and Col2-creERT;DTA^+/+^ mice double stained with Alizarin red and Alcian blue at four weeks of age. Yellow arrow, delayed secondary ossification development in Col2-cre;DTA^+/−^ mice. **i** Quantitative analysis of the femur length and tibia length of 4-week-old Col2-creERT (CreERT) and Col2-creERT;DTA^+/+^ (DTA) mice (*n* = 6 mice per condition; three independent experiments). The data are presented as the means ± s.ds. **j** Total view of the skulls of Col2-creERT and Col2-creERT;DTA^+/+^ mice stained with Alizarin red and Alcian blue at four weeks of age. **k** Total view of the forelimb of Col2-creERT and Col2-creERT;DTA^+/+^ mice stained with Alizarin red and Alcian blue at four weeks of age. **l** Safranin O/Fast Green staining of coronal sections of the femurs of 4-week-old Col2-creERT and Col2-creERT;DTA^+/+^ mice. **m** High-magnification image showing secondary ossification and cartilage in the femurs of 4-week-old Col2-creERT and Col2-creERT;DTA^+/+^ mice. **n** High-magnification picture showing the chondrocyte morphology of 4-week-old Col2-creERT and Col2-creERT;DTA^+/+^ mice. **o** Immunofluorescence staining of the GP and AC of 4-week-old Col2-creERT and Col2-creERT;DTA^+/+^ mice for type 2 collagen. **p** High-magnification image showing type 2 collagen staining in 4-week-old Col2-creERT and Col2-creERT;DTA^+/+^ mice. **q** Phalloidin staining showing the cartilage cytoskeleton of 4-week-old Col2-creERT and Col2-creERT;DTA^+/+^ mice. **r** Quantitative measurements of the percentage of cells with intact actin fragments relative to the total cells in Col2-creERT (CreERT) and Col2-creERT;DTA^+/+^ (DTA) mice (*n* = 6 mice per condition; three independent experiments). The data are presented as the means ± s.ds. The statistical significance was determined by one-way ANOVA and Student’s *t* test. *P < 0.05, **P < 0.01, ***P < 0.000 1, NS not statistically significant

To assess the contribution of Col2^+^ cells to postnatal skeletal development in mice, we genetically ablated these cells by inducing the expression of DTA postnatally in Col2^+^ cells. Specifically, we applied tamoxifen (TM) to either TM-inducible type II collagen Cre (Col2-creERT) or Col2-creERT;DTA^+/+^ mice on postnatal day 3 (P3) and harvested the mice at four weeks of age. All Col2-creERT;DTA^+/+^ mice developed postnatal dwarfism with shorter limbs and body lengths (Fig. 4a–e). Skeletal radiographs and whole-mount Alizarin red staining of the skeleton confirmed this observation and revealed skeletal defects, including epiphyseal dysplasia in the long bone, abnormalities of the capital femoral epiphyses and underdevelopment of the femoral head (Fig. 4d, h). Representative micro-CT images showed epiphyseal dysplasia and substantially decreased bone mass in the long bones of Col2-creERT;DTA^+/+^ mice (Fig. 4f, g). Quantitative analysis revealed that the percentage of bone volume to total bone volume (BV/TV), trabecular number (Tb.N), and trabecular thickness (Tb.Th) of the Col2-creERT;DTA^+/+^ mice were reduced to approximately 0.62-, 0.53-, and 0.5-fold and that the trabecular spacing (Tb.Sp) was increased 1.5-fold compared with those of the Col2-creERT controls (Fig. 4f). Furthermore, Alizarin red/Alcian blue staining showed loss of bone and cartilage in the femur secondary ossification center in 4-week-old Col2-creERT;DTA^+/+^ mice (Fig. 4h). Quantitative analysis demonstrated that the lengths of the femur and tibia bones of the mutant group were significantly shortened to 0.55 cm and 0.78 cm, respectively, compared with the lengths of 1.22 cm and 1.4 cm found in the WT mice (Fig. 4i). Moreover, the postnatal ablation of Col2^+^ cells also impaired skull and long bone and cartilage development (Fig. 4j, k).

### Postnatal deletion of Col2^+^ cells disrupted endochondral ossification and cell alignment

To gain more insight into the skeletal changes, we performed safranin O/fast green staining of tibial sections from 4-week-old Col2-creERT and Col2-creERT;DTA^+/+^ mice (TM injected at P3). The results further confirmed the significantly decreased bone mass in the tibia and the absence of secondary ossification centers in Col2-creERT;DTA^+/+^ mice (Fig. 4i). Higher magnification examination of the secondary ossification center and growth plate (GP) in the tibia of Col2-creERT;DTA^+/+^ mice showed that the GP pattern was substantially disrupted, and the epiphyses were occupied by disorganized hypertrophic chondrocytes^22^ (Fig. 4m, n).

Immunofluorescence staining showed that the type II collagen matrix was enriched in articular cartilage (AC) and GP chondrocytes in 4-week-old WT mice (Fig. 4o, p) and was substantially reduced in Col2-creERT;DTA^+/+^ mice, which indicated that Col2^+^ cells are essential for type II collagen production. Moreover, the F-actin immunostaining results showed that the cell alignment and pattern in GP chondrocytes were markedly disrupted. The quantitative analysis of these results demonstrated that the percentage of cells with intact actin filaments was decreased from 100% in the WT mice to 9.2% in the mutant mice. These findings indicate that the type II collagen matrix and Col2^+^ cells play important roles in cell patterning and GP organization (Fig. 4q, r).

### Spatial distribution of embryonic and postnatal Col2^+^ cells in the long bones and knee joints of mice

To investigate the contribution of Col2^+^ progenitors to skeletal development during the embryonic and postnatal stages, we traced the fate of Col2- expressing cells for different time periods in Col2-cre;tdTomato mice, in which Col2^+^ cells exhibit tomato fluorescence starting from the embryonic stage, and Col2-creERT;tdTomato mice, in which Col2^+^ cells express tomato fluorescence protein after treatment with TM at the indicated postnatal time. These findings demonstrated that Col2^+^ cells from both mouse strains and their descendants were permanently marked by the expression of the red fluorescent protein tdTomato.

**Fig. 5:**
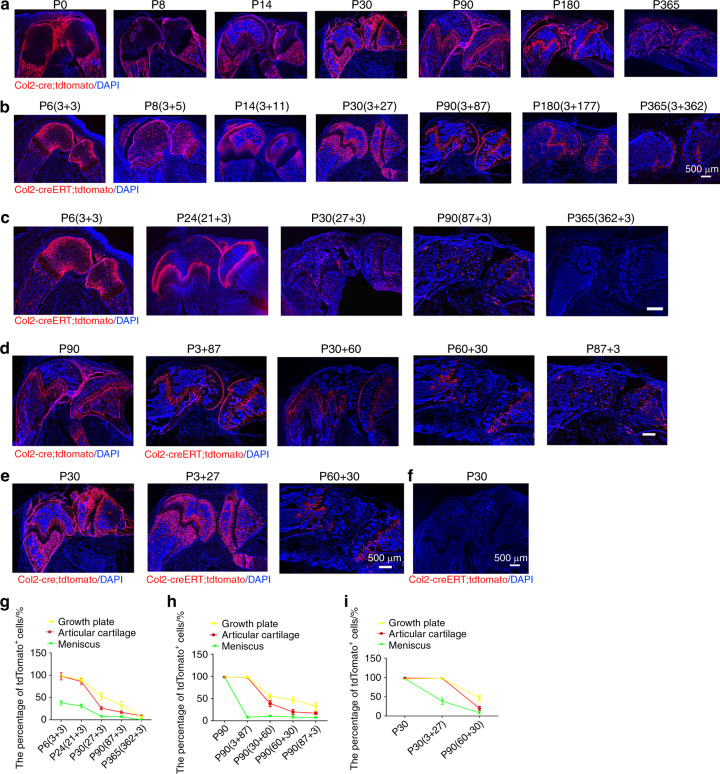
Spatial distribution of embryonic and postnatal Col2^+^ cells in mouse long bones and knee. **a** Representative images from the lineage tracing of embryonic Col2^+^ cells in long bones and knee joints at different time points (P0, P8, P14, P30, P90, P180 and P365). **b** Representative images from the lineage tracing of postnatal Col2^+^ cells in long bones and knee joints at different time points (P6, P8, P14, P30, P90, P180 and P365). This analysis was performed by injecting 75 mg·kg^−1^ tamoxifen into P3 mice. To better present the data, the information for the Col2-creERT;tdTomato mice in each figure is presented as the harvest time (tamoxifen injection date + tracing time period)., e.g., P6 (3 + 3) means that the harvest date of Col2-creERT;tdTomato mice is P6, that tamoxifen was injected at P3 and that tracing was performed for three days. Because all Col2^+^ cells were activated starting from the embryonic stage in Col2-cre;tdTomato mice, we included only the date for the harvesting of mouse samples. For example, P8 means that the harvest date for the Col2-cre;tdTomato mice was P8. **c** Representative images from the lineage tracing of postnatal Col2^+^ cells in long bones and knee joints activated at different time points (P3, P21, P27, P87, and P362). Tamoxifen (75 mg·kg^−1^) was injected into mice at the indicated time points. **d** Representative images from the lineage tracing of Col2^+^ cells in long bones and knee joints at P90 after activation at different time points (embryonic stage, P3, P30, P60 and P87). **e** Representative images from the lineage tracing of Col2^+^ cells in the long bones and knee joints of mice at P30 after activation at the indicated time points (embryonic stage, P3, and P60). This analysis was performed by the injection of 75 mg·kg^−1^ tamoxifen into Col2-creERT;tdTomato mice at different time points. **f** Representative images of the long bones and knee joint of Col2-creERT;tdTomato mice at P30. This analysis was performed by the injection of 75 mg·kg^−1^ vehicle into Col2-creERT;tdTomato mice at P3. **g** Quantitative measurements of the percentage of tdTomato^+^ cells with respect to the total cells in (**c**) (*n* = 6 mice per condition; three independent experiments). The data are presented as the means ± s.ds. **h** Quantitative measurements of the percentage of tdTomato^+^ cells with respect to the total cells in (**d**) (*n* = 6 mice per condition; three independent experiments). The data are presented as the means ± s.ds. **i** Quantitative measurements of the percentage of tdTomato^+^ cells with respect to total cells in (**e**) (*n* = 6 mice per condition; three independent experiments). The data are presented as the means ± s.ds. At least 1 000 cells of each sample were measured. The statistical significance was determined by one-way ANOVA and Student’s *t* test. *P < 0.05, **P < 0.01, ***P < 0.000 1, NS not statistically significant

To examine how Col2^+^ progenitors contribute to skeletal development in Col2-cre;tdTomato mice during the embryonic stage, we first examined the long bones and knee joints and found that tdTomato^+^ cells were present in the AC, GP, bone surface, osteocytes, tendon and meniscus (Fig. 5a). Interestingly, the tdTomato^+^ fluorescence signal was weaker in the GP but stronger in the trabecular and cortical bone and meniscus at P0 and P8. Notably, the tdTomato^+^ fluorescence was stronger in every compartment of the knee at P14 and P30 than at P0. With aging, tdTomato^+^ fluorescence in the trabecular and cortical bone gradually decreased; however, tdTomato^+^ fluorescence in the AC and GP strengthened from P90 to P180 and then decreased at P365.

To further examine the contribution of postnatal Col2^+^ cells to long bone and knee joint development, Col2-creERT;tdTomato mice were intraperitoneally (i.p.) administered TM at P3 and harvested at P6, P8, P14, P30, P90, P180, and P365. At P6 and P8, tdTomato^+^ (Col^+^) cells were predominantly detected in the AC, secondary ossification center, GP, and meniscus (Fig. 5b). Interestingly, at P14 and P30, the pattern of Col2^+^ cells in the AC, GP and long bones of these mice was similar to that found in Col2-cre;tdTomato mice, and this pattern involved decreased tdTomato^+^ fluorescence in the AC and GP but increased fluorescence in the cortical and cancellous bones. At P90, tdTomato^+^ fluorescence was decreased in the cortical and cancellous bones but increased in the GP and AC. However, starting from P180, the numbers of tdTomato^+^ cells in cortical and cancellous bones as well as the GP and AC significantly decreased with aging in both Col2-cre;tdTomato and Col2-creERT;tdTomato mice (Fig. 5a, b).

### The numbers of Col2^+^ progenitors decreased during aging

To further explore the effect of age on the fate of Col2^+^ progenitors, Col2-creERT;tdTomato mice were i.p. injected with TM at P3, P21, P27, P87, and P362, and sections were analyzed 3 days after each injection. As shown in Fig. 5c, tdTomato^+^ cells were detected in articular chondrocytes, the GP and the meniscus after the administration of TM at P3. However, the number of tdTomato^+^ cells in mice significantly decreased during aging. Approximately 98.1% of GP chondrocytes were tdTomato^+^ in the mice administered the TM injection at P3, and the injection of TM at P21, P27, P87, and P362 decreased this percentage to 89.9%, 53%, 32.7%, and 23.2%, respectively. Similarly, approximately 97.8% of articular chondrocytes were tdTomato^+^ in the mice that were injected with TM at P3, and the injection of TM at P21, P27, P87, and P362 decreased these percentages to 86.2%, 26.4%, 17.2%, and 9.1%, respectively (Fig. 5c, g). In the meniscus, 38.1% of cells were tdTomato^+^ in the mice that were injected with TM at P3, and the injection of TM at P21, P27, and P87 decreased this percentage to 31.1%, 7.8%, and 6.9%, respectively, whereas only a few positive cells were detected after the injection of TM at P362. The results demonstrated that the numbers of Col2^+^ progenitors decreased during aging.

To determine how age affects the number and differentiation ability of Col2^+^ cells, we compared the number of tdTomato^+^ cells in the knee joints at P90 between Col2-cre;tdTomato mice whose Col2^+^ cells were activated at the embryonic stage and Col2-creERT;tdTomato mice injected with TM to turn on tdTomato protein expression at P3, P30, P60, and P87 (Fig. 5d). The number of tdTomato^+^ cells gradually decreased from the embryonic stage to P3, P30, P60 and P87. Specifically, at P90, 98.5% of GP chondrocytes were tdTomato^+^ when Col2^+^ was activated at the embryonic stage; however, the percentage of these cells decreased to 98.2%, 55.4%, 47.1%, and 37.2% in the Col2-creERT;tdTomato mice injected with TM at P3, P30, P60 and P87, respectively (Fig. 5D, H). Similarly, approximately 98.4% of articular chondrocytes were tdTomato^+^ when Col2^+^ was activated at the embryonic stage, and the injection of TM at P3, P30, P60 and P87 decreased this percentage to 97.9%, 38.7%, 20.1%, and 17.2%, respectively. In the meniscus, 98.8% of cells were tdTomato^+^ when Col2^+^ was activated at the embryonic stage, and the injection of TM at P3, P30, P60 and P87 decreased this percentage to 8.1%, 10.4%, 8.4%, and 6.9%, respectively. These results suggested that the numbers and differentiation ability of Col2^+^ cells decreased during the aging process.

To further confirm that the differentiation ability of Col2^+^ progenitors decreased with increasing age, we compared the tdTomato^+^ cells in the knee joints over 1 month of tracing among mice whose Col2^+^ cells were activated at the embryonic stage, P3 and P60 (Fig. 5e). Approximately 98.4% of articular chondrocytes were tdTomato^+^ when Col2^+^ was activated at the embryonic stage, and this percentage decreased to 97.9% and 38.7% when TM was injected at P3 and P60, respectively (Fig. 5E, I). Similarly, 98.5% of GP chondrocytes were tdTomato^+^ when Col2^+^ was activated at the embryonic stage, and the injection of TM at P3 and P60 decreased this percentage to 98.2% and 55.4%, respectively. In the meniscus, 98.8% of cells were tdTomato^+^ when Col2^+^ was activated at the embryonic stage, and the injection of TM at P3 and P60 decreased this percentage to 8.1% and 1.3%, respectively (Fig. 5e, i). These results further confirmed that the differentiation ability of Col2^+^ progenitors decreased with age. No detectable td-Tomato fluorescence was detected in the control Col2-creERT;R26-tdTomato mice injected with vehicle, which confirmed the specificity of Cre recombinase activity (Fig. 5f).

In addition, comparing type II collagen expression between embryonic and postnatal Col2^+^ cells revealed that the expression pattern of type II collagen in Col2-cre;tdTomato mice at P6 was similar to the pattern of Col2^+^ cells at P6 in Col2-creERT;tdTomato mice injected with TM at P3 (Fig. S2). However, the type II collagen expression pattern in Col2-cre;tdTomato mice was completely different from the patterns of Col2^+^ cells at P30, P180 and P365 in Col2-creERT;tdTomato mice injected i.p. with TM at P27, P177 or P362 (Fig. S2A–D).

To further demonstrate the differentiation ability of Col2^+^ cells during aging, we performed in vitro primary cell culture. Col2^+^ chondrocytes were harvested from 5-day-, 1-month- and 5-month-old mice, and the differentiation ability was then tested through chondrogenesis and osteogenesis differentiation assays. The results showed that the differentiation potential of Col2^+^ cells decreased with increasing age. A quantitative analysis of the Alizarin red and Alcian blue staining results showed that Col2^+^ chondrocytes from P5 and 1-month-old mice had markedly stronger osteogenic and chondrogenic differentiation potential than Col2^+^ chondrocytes from 5-month-old mice (Fig. S3). These results further indicate that the differentiation ability of Col2^+^ progenitors gradually decreased during aging.

### CD31^+^ cells originating from Col2 progenitors expressed Col2 throughout their lifespan until the time points examined

**Fig. 6:**
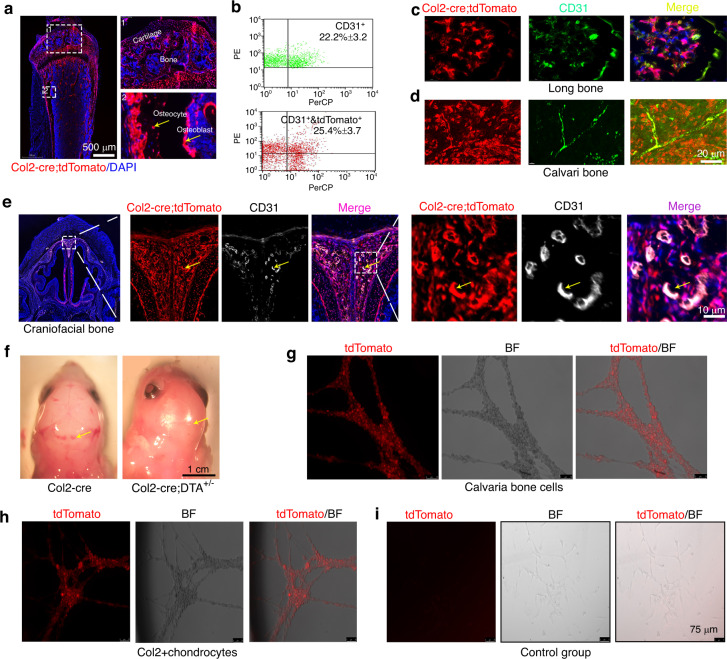
Col2^+^ progenitors have multilineage differentiation ability, including CD31^+^ blood vessels. **a** Representative fluorescent images of the distal femur showing that cartilage cells, osteoblasts and osteocytes are Col2^+^. Yellow arrow, osteoblasts and osteocytes of 4-week-old Col2-cre;tdTomato mice. **b** Flow cytometry analysis was performed using dissociated long bone marrow cells collected from 4-week-old Col2-cre;tdTomato mice. Representative dot plots show that some Col2^+^ bone marrow stromal cells were CD31^+^ (*n* = 3 mice per condition; three independent experiments). **c** Representative fluorescent images of the distal femurs of 4-week-old Col2-cre;tdTomato mice showing that some CD31^+^ cells in long bones were Col2^+^ cells. **d** Representative fluorescent images from the calvarial bone of 4-week-old Col2-cre;tdTomato mice showing that almost all CD31^+^ cells were Col2^+^ cells. **e** Representative fluorescent images from the craniofacial bone of 4-week-old Col2-cre;tdTomato mice showing that almost all CD31^+^ cells were Col2^+^ cells. Yellow arrow, overlapping staining of blood vessels. **f** The gross appearance of the head of Col2-cre and Col2-cre;DTA^+/−^ newborn mice. Note that the mutant mice showed a marked loss of blood vessels in the skull. Yellow arrow, blood vessels in each group. **g** Representative picture showing that Col2^+^ cells from the calvaria bone of Col2-cre;tdTomato mice exhibit tube formation capacity. **h** Representative picture showing that Col2^+^ cells from the cartilage of Col2-cre;tdTomato mice exhibit tube formation capacity. **i** Representative picture showing that Col2^−^ cells from the cartilage of Col2-cre;tdTomato mice do not have tube formation capacity. All the data are reported as the means ± s.ds. The statistical significance was determined by one-way ANOVA and Student’s *t* test. *P < 0.05, **P < 0.01, ***P < 0.000 1. NS not statistically significant

Our results showed that the gross appearance of the Col2-cre;DTA^+/−^ newborn mice exhibited a lack of blood vessel phenotype with pale skin (Fig. 2a, Fig. S1B, C). To further examine whether Col2^+^ cells contribute to blood vessel formation, we first traced the Col2^+^ cell locations in the skeleton of Col2-cre;tdTomato mice and found that Col2^+^ cells (tdTomato^+^) were present in the GP, AC (chondrocytes) and bone (osteoblasts and osteocytes) of 4-week-old Col2-cre;tdTomato mice (Fig. 6a, Fig. S4). Surprisingly, some CD31^+^ cells in blood vessels of the long and skull bones were also labeled with tdTomato^+^ fluorescence (Fig. 6b–f). Flow cytometry results showed that approximately 25.4% of CD31^+^ cells in the long bones of 4-week-old Col2-cre;tdTomato mice were Col2^+^ (Fig. 6b, c), whereas almost all CD31^+^ blood vessel cells in skull bone were Col2^+^ (Fig. 6d). Additionally, many CD31^+^ blood vessel cells in the brain, eyeball, heart, and skin were Col2^+^ (Fig. 6e, Fig. S5A–C), but none of the CD31^+^ cells found in the kidney vasculature during development were Col2^+^ (Fig. S5D). Consistently, we found that the ablation of Col2^+^ cells led to a marked decrease in the numbers of CD31^+^ blood vessels in the skull bone and eyes of Col2-cre;DTA^+/+^;tdTomato mice compared with those detected in the control Col2-cre;tdTomato mice (Fig. 6f, Fig. S5E, F). These results indicate that Col2^+^ cells can contribute to skeletal blood vessel formation.

To further investigate whether Col2^+^ cells could form vascular lumens in vitro, we harvested Col2^+^ cells from the calvarial bone and cartilage of 1-month-old Col2-cre;tdTomato mice and tested the angiogenic tube formation capacity through angiogenesis assays. Indeed, Col2^+^ cells from calvarial bone and cartilage formed vascular lumens in vitro (Fig. 6g–i).

Both hematopoietic and blood vessel endothelial cells are differentiated from hemangioblasts of mesodermal cells.^1^ To determine whether Col2^+^ cells in the bone marrow contribute to hematopoietic cell-derived osteoclast formation, Bone marrow cells(BMCs) from Col2-cre;tdTomato mice were induced with RANKL/M-CSF. As shown in Fig. S6, osteoclasts did not exhibit tdTomato fluorescence. Moreover, TRAP staining for osteoclastogenesis assays confirmed that Col2^+^ cells cannot differentiate into osteoclasts.

### Col2^+^ cells in the GP and AC displayed stem cell properties

**Fig. 7:**
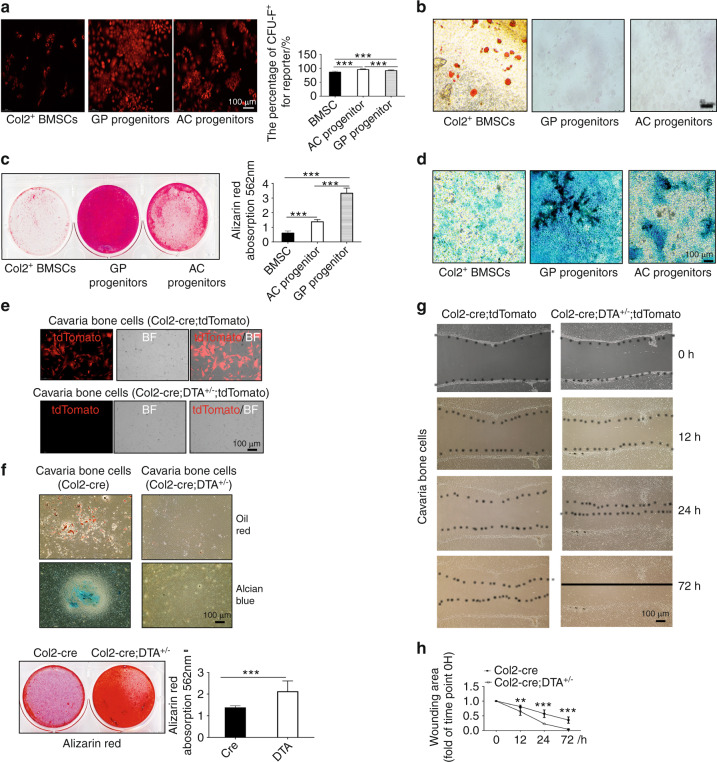
Col2-positive progenitors from different tissues have varied differentiation potential. **a** CFU-F assay of Col2^+^ bone marrow stem cells (BMSCs), GP progenitors and AC progenitors showing that every cell type can form CFU colonies. **b** Adipogenic differentiation of Col2^+^ BMSCs, GP progenitors and AC progenitors. **c** Osteogenic differentiation of Col2^+^ BMSCs, GP progenitors and AC progenitors. **d** Chondrogenic differentiation of Col2^+^ BMSCs, GP progenitors and AC progenitors. **e** Merged pictures showing Col2^+^ cells cultured from the calvaria of Col2-cre;tdTomato and Col2-cre;DTA^+/−^;tdTomato newborns. Note that few Col2^+^ cells were detected in the Col2^+^ cell ablation group, confirming the effectiveness of the cell ablation technique. **f** Assay of the trilineage differentiation of cells cultured from the calvarial bone of Col2-cre and Col2-cre;DTA^+/−^ newborns (*n* = 3 mice per condition; three independent experiments). **g** Different migration abilities of cells cultured from Col2-cre and Col2-cre;DTA^+/−^ newborns. **h** Quantitative measurements of migration ability based on the results shown in (**g**) (*n* = 3 mice per condition; three independent experiments). All the data are reported as the means ± s.ds. The statistical significance was determined by one-way ANOVA and Student’s *t* test. *P < 0.05, **P < 0.01, ***P < 0.000 1, NS not statistically significant

Our results showed that Col2^+^ cells were located in the AC, bone marrow and GP and that the ablation of Col2^+^ cells completely disrupted skeletal development with the exception of craniofacial bone development. To determine whether Col2^+^ cells exhibit stem cell properties, we first performed a CFU-F activity assay. BMCs, GP chondrocytes and articular chondrocytes were isolated from 4-week-old Col2-cre;tdTomato mice, and the Col2^+^ cells were then sorted, as shown in Fig. 7a. The quantification of tdTomato^+^ CFU-F colonies revealed that Col2^+^ cells from BMCs, GP chondrocytes and articular chondrocytes could all form CFU-F colonies (Fig. 7a).

To further assess whether Col2^+^ cells have multiple lineage differentiation capabilities, Col2^+^ cells from the bone marrow, GP and AC were induced with osteogenic, chondrogenic and adipogenic media for the indicated times (Fig. 7b). Interestingly, Col2^+^ cells sorted from BMCs could differentiate into osteoblasts, chondrocytes and adipocytes, whereas Col2^+^ cells from the GP and AC could differentiate only into osteoblasts and chondrocytes but not into adipocytes (Fig. 7b–d). These findings suggested that Col2^+^ cells from BMCs are likely bone mesenchymal stem cells (BMSCs) at earlier stages, whereas a large population of Col2^+^ cells from the GP and AC were BMSCs at later stages. Most interestingly, the Alcian blue and Alizarin red staining of the cells showed that Col2^+^ cells from the GP exhibited markedly higher osteogenic and chondrogenic differentiation potential than Col2^+^ cells from articular chondrocytes and BMCs (Fig. 7c, d).

Oct4 and Sox2 are stem cell markers.^23,24^ To determine whether Col2^+^ cells express stem cell markers, we coimmunostained sections of the GP, bone marrow and AC tissues from newborn and 1-month-old Col2-cre;tdTomato mice to detect the expression of Oct4 and Sox2. Our data showed that Oct4 and Sox2 expression overlapped with Col2^+^ cells in the bone marrow, GP and AC (Fig. S7A), which confirmed that Col2^+^ cells from these three sources have stem cell properties. Moreover, our data showed that Oct4- and Sox2-positive cells overlapped with some Col2^+^ cells in the bone marrow but with markedly fewer Col2^+^ cells in the GP and AC than in the bone marrow of Col2-cre;tdTomato mice (Fig. S7B, C).

### Col2-negative cells from the calvarial bone but not cartilage show high unipotent osteogenic potential

According to the lineage tracing results shown in Fig. 3c, both Col2^+^ and Col2^−^ cells are present in calvarial bone. To further test whether Col2^−^ cells also have differentiation potential, calvarial bone cells were isolated from the calvaria of newborn Col2-cre;tdTomato (control) and Col2-cre;DTA^+/−^;tdTomato mice. We found that approximately 50% of cells in the control group were Col2^+^, but almost no Col2^+^ cells were detected in the Col2-cre;DTA^+/−^;tdTomato group, which confirmed the effectiveness of Co2^+^ cell ablation (Fig. 7e). Moreover, we found that the cells of the control calvarial bone could differentiate into three lineages, namely, osteoblasts, chondrocytes and adipocytes. Interestingly, Col2^−^ cells could not differentiate into chondrocytes and adipocytes but exhibited markedly higher potential to differentiate into osteoblasts than control cells (Fig. 7f). Additionally, the wound healing assay showed that Col2^−^ cells had stronger migration ability than the control cells (Fig. 7g, h).

To investigate whether the differentiation potential differs between Col2^+^ and Col2^−^ cells, we isolated Col2^+^ and Col2^−^ cells from the bone marrow of the long bones of 1-month-old Col2-cre;tdTomato mice. We found that both Col2^+^ and Col2^−^ cells were capable of differentiating into osteoblasts and chondrocytes (Fig. S8). A quantitative analysis of Alcian blue- and Alizarin red-stained cells showed that Col2^+^ cells from the bone marrow have markedly higher osteogenic and chondrogenic differentiation potential than Col2^−^cells (Fig. S8).

### Col2^+^ progenitors contributed to chondrocyte differentiation and blood vessel formation during fracture healing

**Fig. 8:**
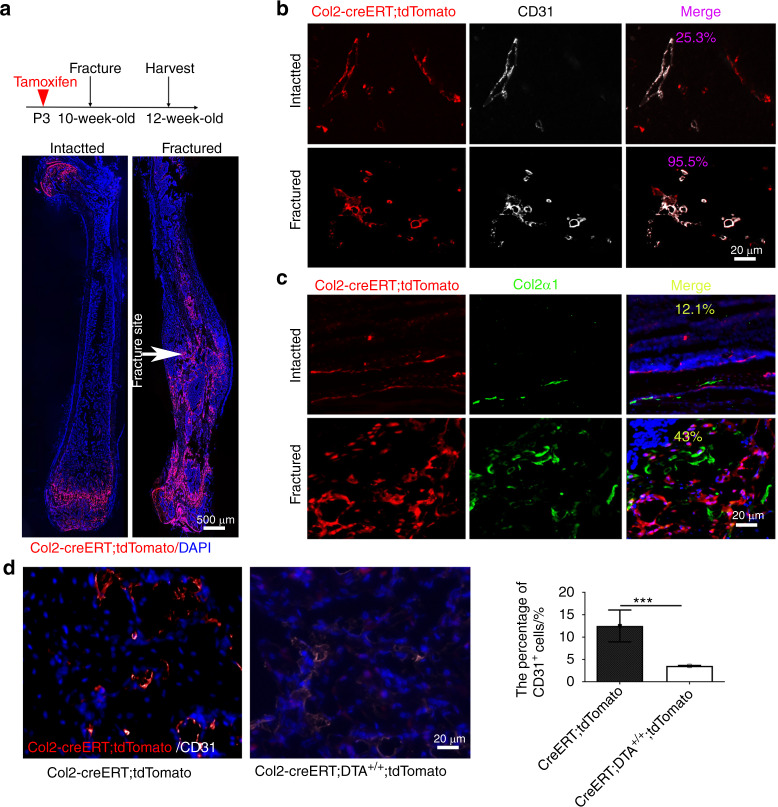
Col2^+^ progenitors contribute to chondrocyte differentiation and blood vessel formation during fracture healing. **a** Illustration of the experimental design and representative images of femoral sections of control and fractured Col2-creERT;tdTomato mice. This analysis was performed by injecting 75 mg·kg^−1^ tamoxifen into mice at P3, subjecting the mice to fracture at 10 weeks of age, and harvesting the tissues after two weeks of healing. **b** Representative confocal images of femoral sections from the intact and fracture groups (post-injury) through the fracture site with immunofluorescence staining for CD31 (white) to demarcate the blood vessels (*n* = 3 mice per condition; three independent experiments). White arrow, fracture site. **c** Representative confocal images of femoral sections of the intact and fracture groups (postinjury) through the fracture site with immunofluorescence staining for CD31 (white) to demarcate chondrocytes (*n* = 3 mice per condition; three independent experiments). **d** Representative confocal images of femoral sections through the fracture site of Col2-creERT;tdTomato and Col2-creERT;DTA^+/+^;tdTomato mice with immunofluorescence staining for CD31 (white) to demarcate the blood vessels in^+/+^ (*n* = 3 mice per condition; three independent experiments). All the data are reported as the means ± s.ds. The statistical significance was determined by one-way ANOVA and Student’s *t* test. *P < 0.05, **P < 0.01, ***P < 0.000 1, NS not statistically significant

To test whether Col2^+^ cells contribute to postnatal chondrocyte differentiation and blood vessel formation, we created a closed femoral fracture with an intramedullary nail fixation model in 10-week-old mice as previously described^25^ to trace Col2^+^ cells during fracture healing. We first induced tdTomato expression in Col2^+^ cells of Col2-creERT; tdTomato mice through the injection of TM at P3, induced a fracture in the mice at 10 weeks of age and harvested tissues two weeks after the fracture surgery. In the noninjured mice, the contralateral nonfractured femur exhibited prominent tdTomato^+^ cells in the AC and metaphysis but few tdTomato^+^ cells in the bone marrow (Fig. 8a). However, strong tdTomato expression was detected throughout the fractured callus, including both bony and cartilaginous regions (Fig. 8b, c). Immunofluorescent staining for CD31 and Col2α1 showed that 95.5% of the CD31^+^ cells and 43% of the Col2α1^+^ cells overlapped with tdTomato^+^ (Col2^+^) cells in the callus area of Col2-creERT;tdTomato mice. In contrast, only 25.3% of CD31^+^ cells and 12.1% of Col2α1^+^ cells overlapped with tdTomato^+^ (Col2^+^) cells in the intact long bones of Col2-creERT;tdTomato mice (Fig. 8b, c). Immunofluorescent staining for blood vessels in the fractured callus showed that the percentage of CD31^+^ cells decreased markedly to 3.5% in Col2-creERT;DTA^+/+^;tdTomato mice compared with 12.5% in Col2-creERT;tdTomato mice (Fig. 8d). These results indicate that Col2^+^ cells play an important role in blood vessel and bone regeneration during fracture healing.

## Discussion

Embryonic type II collagen-positive (Col2^+^) cells reportedly represent an early mesenchymal progenitor that contributes to endochondral ossification.^6,19^ The findings from this study provide the first demonstration that ablation of Col2^+^ progenitors starting at the embryonic stage causes lethality in newborn mice due to dyspnea and the absence of a skeleton. In contrast to the previous understanding that only flat bone is ossified intramembranously, we found that the ablation of Col2^+^ cells impaired calvarial bone development and led to complete loss of vertebral bone development and most of the long bone and cartilage. In addition, we found that Col2^+^ cells contribute to blood vessel formation in bone tissue and some other organs, including the brain, heart and skin. As mesenchymal cells differentiate into immature chondrocytes, which are marked by type II collagen,^26^ at embryonic day 13.5 (E13.5), all the tdTomato^+^ cells (including those in blood vessels and other tdTomato^+^ cells) found at later stages should originate from cartilage (chondrocytes) at E13.5. Furthermore, our long-term fate mapping study revealed that postnatal Col2^+^ progenitors existed in the AC as well as in the bone marrow and GP and that the numbers and differentiation ability of these Col2^+^ progenitors decreased with increasing age.

The bones of the cranial base and caudal cranial vault ossify endochondrally, whereas the facial bone and rostral cranial vault ossify intramembranously.^1,27,28^ Consistently, our results showed that Col2-negative cells exist in most craniofacial bones. However, in contrast to previous reports,^1,29,30^ we also found Col2^+^ cells in craniofacial flat bones, including the mandible, premaxilla, parietal, frontal, jugal, palatine and temporal bones and clavicle, and participated in their development (Fig. S9), and the ablation of Col2^+^ cells affected the formation of these bones. Endochondral and intramembranous ossification are two processes involved in bone development in humans and mice. Because Col2^+^ cells contribute to endochondral ossification,^4,6^ ablation of Col2^+^ cells should remove all endochondral ossifications. Moreover, the bones that consist of Col2^−^ cells should be intramembranously ossified. Thus, our findings suggest that the endochondral ossification process may also contribute to skull bone development. This conclusion is supported by the findings reported by Akagami N et al.,^21^ who found that a large number of osteoblasts and suture mesenchymal cells in the calvaria express Col2. Similarly, the ribs, long bones and vertebral bones were previously reported to ossify endochondrally during development^1,2,31^; however, we found that the ablation of Col2^+^ cells did not completely impair rib and long bone formation but caused complete loss of vertebral bone. These findings indicate that ribs and long bones may be ossified both intramembranously and endochondrally, that vertebral bone ossifies purely endochondrally, and that Col2^+^ cells are the major stem cells that drive vertebral development. During the spine and vertebral development process, Col2^+^ cells were first detected at E9.5 in the mouse sclerotome and notochord,^32,33^ demonstrating that resident Col2^+^ populations develop from E9 to E9.5. Between E11 and 12, the unsegmented perichondral mesenchyme differentiates into the annulus fibrosus of the disc anlagen. Meanwhile, uncondensed mesenchyme develops into cartilaginous vertebral bodies. Starting from E14.5, the annulus is divided into fibrosus outer and cartilaginous inner parts, and then the inner annulus links the disc rudiments to the developing vertebral bodies.^34^ Moreover, the ossification marker Cbfa1 was first detected at approximately E12.5 in condensed cartilage.^35^ Rigueur D et al. confirmed that the vertebral column displays positive Alcian blue staining (cartilage) and is negative for Alizarin red staining at E14.5 (bone) but positive in a small area of E16.5 mouse embryosstaining.^36^ Our data showed that the vertebral column was ossified at E17.5, and stage-specific ablation of Col2 cells at E17.5 resulted in loss of the vertebral column (Fig. 2c). Thus, these results indicate that ossification of the vertebral column most likely commences at E15 or E16.^6^ Based on the above findings, we speculate that Col2^+^ populations can contribute to multiple cell types in the spine, including vertebral cells and intervertebral disc cells.^37^ Thus, ablation of Col2^+^ cells starting from E9.5 completely disrupts axial vertebral and intervertebral disc development.

The critical role of angiogenesis during endochondral and intramembranous ossification has been well established.^1^ During endochondral ossification of long bone, the vasculature enters the avascular buffer layer of loose mesenchymal cells surrounding the mid-diaphysis of the cartilage model immediately before the initiation of angiogenesis and the mineralization of a bone collar within the perichondrium. The process of angiogenesis during endochondral ossification of long bones has also been well established, but the corresponding process during intramembranous ossification is generally assumed. Despite some similarities between calvarial and long bone osteogenesis in vascular invasion into the surrounding avascular loose mesenchyme and the association of invading vessels with mineralization, the fundamental difference in the osteogenesis of endochondral and intramembranous bones provides hints regarding the hypothetical differences in angiogenesis between the two types of bone formation processes.^38^ In agreement with this theory, we found that almost all Col2^+^ cells in the blood vessels of skull bone were CD31^+^, but only 25.4% of CD31^+^ cells in long bone were Col2^+^ cells, which suggested different cell origins for blood vessel formation in skull and long bones (Fig. S10).

Blood vessels are composed of several different cell types. The inner layer of blood vessels is composed of endothelial cells (ECs), which are covered on the outer abluminal surface by perivascular (or mural) cells.^38^ Angiogenesis requires extensive coordination among the different vascular cell types to ensure that new vessels are fully functional and stable. An increasing body of evidence indicates that blood vessels form and become specialized in an organ-specific manner and that this process is controlled by local microenvironmental signals, leading to specific molecular signatures in Ecs.^39^ Through lineage tracing of Sox10^+^ cells, Wang et al.^39^ first found that Sox10^+^ cells contribute to only a small number of blood vessels in the lung, spleen and kidney and do not contribute to the liver vasculature during normal development. Consistently, our results showed that Col2^+^ cells contribute differently to CD31^+^ blood vessels in different organs or tissues. Notably, all CD31^+^ cells in blood vessels in the brain, eyeball, heart, skin and skull bone were Col2^+^ cells (Fig. 6D, E, Fig. S3). However, approximately 25.4% of CD31^+^ cells in long bones were Col2^+^ cells, whereas no CD31^+^ cells in the kidney vasculature were Col2^+^ cells during development. Interestingly, although our results suggest that the number of Col2^+^ blood vessels in long bones during normal development is lower than that in skull bones, the number of Col2^+^ blood vessels is markedly increased upon cell expansion following fracture.^39^ Our findings thus provide the first demonstration that blood vessels in the skeleton partially originate from Col2^+^ progenitors.

SSCs are somatic stem cells that play important roles in the development, homeostasis, and regeneration of bone tissues.^40^ These cells are generally defined as self-renewing cells with “trilineage” potential to differentiate into chondrocytes, osteoblasts and adipocytes. SSCs can be classified into three types depending on their location: bone marrow-derived SSCs, GP (a cartilaginous structure separating the primary and secondary ossification centers in growing bones) SSCs and perichondrial/periosteal (the connective tissue surrounding bone) SSCs.^12,41^ Previous studies have demonstrated that Col2^+^ cells behave as SSCs and are present in the bone marrow, GP and perichondrium/periosteum.^12,42^ Interestingly, we found that similar to Pthrp^+^ cells,^43^ Col2^+^ SSCs can differentiate into osteoblasts and chondrocytes but rarely differentiate into adipocytes in vitro. Most interestingly, Col2^+^ cells in the GP have stronger osteogenic and chondrogenic differentiation ability than those in the AC and bone marrow. In addition to these Col2^+^ cells, we also found Col2^+^ cells among calvaria-derived cells.^44^ Interestingly, calvaria-derived Col2^+^ progenitors from Col2-cre;tdTomato control mice can differentiate into osteoblasts, chondrocytes, and adipocytes. However, Col2^−^ cells from the Col2-cre;DTA^+/−^ mouse calvaria can differentiate only into osteoblasts. These results suggest that Col2^+^ cells in the calvaria might be progenitor cells with multipotent differentiation ability. Surprisingly, the comparison of Col2^−^ cells from Col2-cre;DTA^+/−^ and Col2^+^ cells from Col2-cre mice revealed that Col2− cells have a stronger osteogenic ability than Col2^+^ cells, and this finding suggests that Col2^−^cells may originate from neural crest cell lineages, which have been proven to have stronger potential for osteogenesis.^1,45,46^ To further determine the characteristics of Col2^+^ cells and Col2^−^cells, we isolated Col2^+^ and Col2^−^ cells from long bone marrow cells. Interestingly, we found that both Col2^+^ and Col2^−^ cells from bone marrow cells have osteogenic and chondrogenic differentiation potential. However, quantitative analysis showed that Col2^+^ cells have much stronger osteogenic and chondrogenic differentiation potential than Col2^−^ cells from 1-month-old mice. Further study would establish the relationship between Col2^−^ and Col2^+^ progenitors in the calvaria and long bone marrow, which is of interest. In addition, our data further showed that Col2^+^ cells have diverse roles in blood vessel formation between long bone and calvaria bone. Almost all Col2^+^ cells in calvaria bone contribute to blood vessel formation, but only 25.4% of Col2^+^ cells in bone marrow contribute to blood vessel formation. Further investigation will deeply analyze the precise contribution and mechanism of Col2^+^ cells to skeletal blood vessel formation in different bones.

The comparison of the differentiation potential of Col2^+^ cells from the bone marrow, GP and AC showed that bone marrow Col2^+^ cells have osteogenic, chondrogenic and adipogenic differentiation potential. However, Col2^+^ cells in the GP and AC have a markedly stronger capability for osteogenic differentiation than Col2^+^ cells from the bone marrow under the same differentiation conditions. One possible reason is that Col2^+^ cells in the bone marrow are SSCs at early stages, whereas Col2^+^ cells in the GP and AC are stem cells at later stages; thus, under comparable differentiation conditions, Col2^+^ cells in the bone marrow result in less osteoblast and chondrocyte formation than committed osteoblastic and chondrogenic precursor cells. Additionally, a very limited number of Col2^+^ cells are found in the bone marrow after sorting through flow cytometry. To increase the cell numbers, we collected 10x-fold more cells from the bone marrow than from the GP and AC. Thus, the sorting of these cells took a long time. As a result, to obtain sufficient numbers of cells, Col2^+^ cells from the bone marrow were cultured for a longer time than those from the GP and AC, which may affect their stem cell differentiation potential. Further study will interestingly identify the features of these different SSCs by single-cell RNA sequencing.

Our results showed that the numbers of Col2^+^ cells among articular chondrocytes decreased in an age-dependent manner, and this finding is supported by the fact that the numbers of pthrpr1^+^ columns in the GP gradually decreased with aging.^43^ Interestingly, we further found that the Col2^+^ progenitors did not exhibit a decrease at a constant rate during aging; instead, Col2^+^ progenitors decreased rapidly during 2–3 weeks of age and then slowly decreased at later stages. The marked decrease in Col2^+^ cells from 2 to 3 weeks of age may result from genetic changes or the activation/deactivation of hormones/growth factors occurring during this stage. Comparing the gene expression changes in cells between 2 and 3 weeks with those between earlier and later stages could provide information on the mechanisms contributing to bone and cartilage regeneration and repair and age-related osteoarthritis.

Taken together, the results showed that Col2^+^ cells are involved in the development of calvarial flat bone and long bone. Col2^+^ cells are major progenitors that contribute to CD31^+^ blood vessel development in calvarial bone, long bone development and fracture repair. The number and differentiation ability of Col2^+^ progenitors in mice decrease with increasing age. The identification of Col2^+^ and/or Col2^−^ progenitors from the GP, AC and calvarial bone provides new promising therapeutic strategies for bone regeneration and repair and for the treatment of bone and cartilage diseases.

## Methods and materials

### Mice

All procedures regarding mouse housing, breeding, and collection of animal tissues were performed according to protocols approved by the Institutional Animal Care and Use Committee (IACUC) of the University of Pennsylvania in accordance with the relevant guidelines and regulations established by the IACUC. All animals were of the C57BL strain, and all mice were housed under specific pathogen-free conditions. Col2-cre,^47^ Col2-creERT,^48^ R26-tdTomato,^49^ and DTA^+/+^^50^ mice were obtained from Jackson Laboratory (Bar Harbor, ME, USA). Col2-cre; DTA^+/−^ and Col2-creERT; DTA^+/+^ mice were generated by breeding DTA^+/+^ mice with Col2-cre or Col2-creERT mice. Col2-creERT;DTA^+/+^ mice were injected with TM at the indicated time points to induce the postnatal deletion of Col2^+^ cells. To monitor the topography of Col2-cre and Col2-creERT function, we crossed Col2-creERT and Col2-cre mice with R26-tdTomato reporter mice and analyzed the patterns of tdTomato labeling in long bones, cartilage, and knee joints at different time points. The mice were euthanized by an overdose of carbon dioxide. TM (T5648, Sigma) solution was prepared and administered as previously described.^51^ Briefly, TM was first dissolved in 100% ethanol (100 mg·mL^−1^) and then diluted with sterile corn oil to a final concentration of 10 mg·mL^−1^. The TM-oil mixture was stored at 4 °C until use. The mice in both the control and experimental groups were administered the same dose of TM (75 mg·kg^−1^ body weight) according to the time points defined in the experimental protocol.

### Histology

Samples were dissected under a stereomicroscope to remove soft tissues, fixed in 4% paraformaldehyde overnight at 4 °C, and then decalcified in 10% ethylenediaminetetraacetic acid (EDTA) for 14 days at 4 °C. The decalcified samples were cryoprotected in 30% sucrose/PBS solution and then in 30% sucrose/PBS:OCT (1:1) solution overnight at 4 °C. The samples were then embedded in an OCT compound (4583, Sakura) under a stereomicroscope and transferred to a sheet of dry ice to solidify the compound. The embedded samples were cryosectioned at 6 μm using a cryostat (CM1850, Leica).^52^

### Safranin O/fast green staining

The mouse tibia was sectioned and stained with safranin O/fast green to visualize cartilage and assess the proteoglycan content, as described previously.^53^ The samples on the slides were stained with Weigert’s iron hematoxylin and fast green and then with 0.1% safranin O solution.

### Alizarin red/Alcian blue staining

The whole skeleton was subjected to Alizarin red/Alcian blue staining as reported previously.^51^ Briefly, the skeletons of newborn mice (*n* = 3) were fixed with 90% ethanol and then stained with 0.01% Alcian blue solution (26385–01, Electron Microscopy Sciences) and 1% Alizarin red S solution (A47503, Thomas Scientific). The stained skeletons were stored in glycerol.

### Von Kossa staining

Von Kossa staining was performed with 1% silver nitrate solution (LC227501, Fisher Scientific) in a glass Coplin jar placed under ultraviolet light for 20–30 min.^54^ Unreacted silver was washed with 5% sodium thiosulfate (01525, Chem-Impex). The slides were dehydrated through a graded alcohol series and mounted with permanent mounting medium.

### Immunofluorescence microscopy

Tibial sections with a thickness of 6 μm were gently rinsed with PBS and incubated with proteinase K (20 μg·mL^−1^, D3001-2-5, Zymo Research) for 10 min at room temperature. Subsequently, the sections were blocked in 5% normal serum (10000 C, Thermo Fisher Scientific) in PBS-T (0.4% Triton X-100 in PBS) or incubated with antibodies against type II collagen (1:100, ab34712, Abcam), Sox2 (1:200, 66411-1-Ig, Proteintech), Oct4 (1:200, 11263-1-AP, Proteintech) and CD31 (1:100, sc-81158, Santa Cruz Biotechnology) in blocking buffer at 4 °C overnight. The tissue sections were washed three times with PBS and then incubated with Alexa Fluor 488-conjugated anti-rabbit (1:200, A11008, Invitrogen) and Alexa Fluor 647-conjugated anti-mouse (1:200, A-21236, Invitrogen) secondary antibodies for 1 h at room temperature. The coverslips were mounted with FluoroShield (F6057, Sigma–Aldrich).

To quantify the percentage of tdTomato^+^ cells, multiple fields of z-stacked photographs were randomly captured. At least 30 images were measured. The percentage of tdTomato^+^ cells was calculated from the ratio of tdTomato^+^ cells to total cells observed in each compartment and each sample (five sections were collected from each sample). Six mice from each group were evaluated. The assessments were independently performed by two authors who were blinded to the groups.^55^ The average percentage of tdTomato^+^ cells in each sample was pooled and calculated by two authors. The average tdTomato^+^ percentage in each group was obtained from a pool of six mice and calculated.

### PEGASOS passive immersion and blood vessel staining

PEGASOS passive immersion and blood vessel staining were performed as reported previously.^56^ Briefly, before transcardial perfusion, the mice were anesthetized with an intraperitoneal injection of a combination of xylazine and ketamine (10–12.5 mg·kg^−1^ xylazine; 80–100 mg·kg^−1^ body weight ketamine). Subsequently, 50 mL of ice-cold heparin PBS (10 U·mL^−1^ heparin sodium in 0.01 mol·L^−1^ PBS) was injected transcardially to wash out the blood, and 50 mL of 4% paraformaldehyde (PFA) in 0.01 mol·L^−1^ PBS (pH 7.4) was infused transcardially for fixation.

To clear the calvarial bones, the samples were fixed in 4% PFA at room temperature for 12 h and then immersed in 0.5 mol·L^−1^ EDTA (pH 7.0) at 37 °C in a shaker for two days. The samples were then decolorized with Quadrol decolorization solution for one day at 37 °C in a shaker. The samples were subsequently transferred into 1.5-mL Eppendorf tubes containing blocking solution composed of 10% dimethyl sulfoxide, 0.5% IgePal630 and 1X casein buffer in 1 mL of 0.01 mol·L^−1^ PBS overnight blocking at room temperature. After blocking, the samples were stained with CD-31 primary antibodies in blocking solution for three days at 4 °C on a shaker, then washed at least three times with PBS at room temperature for one day. The samples were incubated with freshly prepared secondary antibodies diluted with blocking solution for another three days at 4 °C on a shaker and washed again for half a day, then incubated with 30%, 50%, and 70% gradient tB delipidation solutions for 4 h with each solution and then with tB-PEG for two days for dehydration. The samples were subsequently immersed in BB-PEG medium at 37 °C for half a day for clearing. Images were acquired using an Sp8 confocal microscope (Leica) with a 25X lens. 3-D reconstruction images were generated using Imaris 9.0 (Bitplane). Stacked images were generated using the “volume rendering” function. Optical slices were obtained using the “orthoslicer” function, and 3-D images were generated using the “snapshot” function.

### Micro-CT analysis

A quantitative analysis of the gross bone morphology and microarchitecture was performed by micro-CT (Micro-CT 35, Scanco Medical AG, Brüttisellen, Switzerland) at Penn Center for Musculoskeletal Disorders (PCMD), University of Pennsylvania. Briefly, femurs from 4-week-old Col2-creERT and Col2-creERT; DTA^+/+^ mice were fixed and scanned, and three-dimensional images were reconstituted from the scans. The cross-sectional scans were analyzed to evaluate the changes in the femur. The ROIs were then compiled into 3D datasets using a Gaussian filter (sigma = 1.2, support = 2) to reduce noise and converted to binary images with a fixed grayscale threshold of 316. The trabecular bone architecture between the distal femoral metaphysis and midshaft was assessed. Two hundred (200) slices (2 mm) above the highest point of the GP were contoured for trabecular bone analysis of the BV·TV^−1^, Tb.Th, Tb.N, Tb.Sp.

### Cell culture

Cells were isolated from the calvarial bone of newborns as described previously.^44^ Briefly, calvarial bone was dissected from newborns, subjected to sequential collagenase type II (2 mg·mL^−1^) (17101015, Gibco) and trypsin (SH3004202, GE Healthcare) digestion for 30 min and cut into tiny pieces. The minced bones were treated with collagenase type II and trypsin again and then plated in tissue culture dishes. The cells that migrated from the bone pieces were passaged and used for further studies.

BMSCs were isolated as previously described.^44^ Briefly, femurs and tibias were dissected from 4-week-old Col2-cre;tdTomato mice. The bones were cut on both ends with sterile blades (22079690, Fisher Scientific). The bone marrow was flushed with complete α-MEM using 23-gauge needle syringes. The cells were then transferred for flow cytometry sorting and cell culture.

GP chondrocytes were isolated as previously described.^43^ Briefly, distal epiphyses of the femurs from 4-week-old Col2-cre;tdTomato were manually dislodged, and the attached soft tissues and woven bones were carefully removed. Dissected epiphyses were incubated in 3 mL of Hank’s balanced salt solution (HBSS, H6648, Sigma) at 37 °C for 60 min in a shaking incubator. The AC and secondary ossification centers were subsequently removed. The dissected GPs were minced using a disposable scalpel (22079690, Fisher Scientific) and further incubated with Liberase TM at 37 °C for 60 min in a shaking incubator. The cells were mechanically triturated using an 18-gauge needle and a 1-mL Luer-Lok syringe and filtered through a 70-µm cell strainer into a 50-mL tube on ice to obtain a single-cell suspension. The cells were pelleted and resuspended in the appropriate medium for subsequent analyses.

AC chondrocytes were isolated as previously described.^25^ Briefly, 4-week-old Col2-cre;tdTomato mice were euthanized at P28. The AC from the femoral heads was isolated by first thoroughly removing the soft tissue and bone and then incubating the sample with collagenase type IV (LS004188, Worthington Biochemical Corp) (3 mg·mL^−1^) for 45 min at 37 °C. Cartilage pieces were obtained and incubated in 0.5 mg·mL^−1^ collagenase type IV solution overnight at 37 °C. The cells were then filtered through a 40-μm cell strainer, collected and obtained as a single-cell suspension. The cells were pelleted and resuspended in the appropriate medium for subsequent analyses.

### Flow cytometry

Cells were resuspended in 100 mL of cell staining buffer (420201, BioLegend), incubated with anti-CD16/32 antibody for 20 min on ice to block Fc receptors, and stained with fluorochrome-conjugated or isotype control antibodies on ice for 20 min. To identify MSCs, the cells were incubated with anti-CD31-APC (102419, BioLegend, 1:100) antibody and then stained with biotin-conjugated antibodies. After washing with staining medium, the cells were incubated with streptavidin-brilliant violet 421 TM (BioLegend, 1:500). Flow cytometry analysis was performed using a five-laser BD LSR Fortessa (Ex. 405/488/561/640 nm) and FACSDiva software. The acquired raw data were further analyzed using FlowJo software (Tree Star). Representative plots of at least three independent biological samples are shown in the figure.

### CFU-F assay and in vitro differentiation

The following procedures were modified from a previous report.^57^ For CFU-F assays, freshly prepared unfractionated bone marrow single-cell suspensions were plated at a density of ~10^4^ cells per cm^2^ (0.21 mL of culture medium) in 100-mm dishes containing DMEM supplemented with 20% qualified FBS, 10 mol·L^−1^ Y-27632 (TOCRIS) and 1% penicillin/streptomycin. For CFU-F assays with sorted cells, tdTomato^+^ cells were sorted and directly seeded into culture at a density of 10 cells per cm^2^ in 6-well plates to ensure that colonies would form at clonal density and could thus be counted. The cultures were incubated at 37 °C in a humidified atmosphere with 5% O_2_ and 10% CO_2_ for 7–10 days. The CFU-F colonies were counted after 7–10 days of culture.

For the in vitro differentiation assay, tdTomato^+^ cells were sorted, seeded into each well of 48-well plates and cultured for 14 days. Adipocyte, chondrocyte and osteoblastic differentiation was induced with different differentiation media and detected by staining with oil red O (O0625–25G, Sigma–Aldrich), Alcian blue (26385–01, Electron Microscopy Sciences) and Alizarin red (A47503, Thomas Scientific), respectively, as performed previously.^58,59^

### Bone fracture

Closed femoral fractures with intramedullary nail fixation were created in 10-week-old mice (Col2-creERT;tdTomato or Col2-creERT;DTA^+/+^;tdTomato) as previously described.^25^ Briefly, closed fractures were generated using a three-point blunt guillotine driven by a dropped weight, which created a uniform transverse fracture of the femur. The fractured femurs were harvested at 12 weeks of age for analysis. Three independent experiments with six (*n* = 6) mice per condition were performed.

### Scratch-wound assay

Scratch wound assays were performed as previously described.^60^ The same numbers of cells from the calvarial bone of Col2-cre;tdTomato and Col2-cre;DTA^+/+^;tdTomato mice were seeded in six-well plates. The monolayer cells were scratched and then cultivated under normal conditions. For each group, the migration distances at 0, 12, 24, and 72 h after scratching were measured.

### Blood vessel tube formation assay

Mouse tdTomato^+^ and tdTomato^−^ cells were isolated from the calvarial bone and cartilage of Col2-cre;tdTomato mice and depleted of erythrocytes by hypotonic lysis. tdTomato^+^ and tdTomato^−^ cells were isolated by cell sorting using a BD FACS ARIA II sorter, and the purity of the sorted cells was higher than 95%. The cells were resuspended (1.5 × 10^6^ cells per mL) in endothelial growth medium (EGM-2) supplemented with EGF, hydrocortisone, VEGF, fibroblast growth factor-B, heparin, insulin-like growth factor, gentamicin, and 5% heat-inactivated fetal bovine serum (CC3162, Lonza).

To evaluate the effect of tdTomato^+^ and tdTomato^+^ cells on tube formation capacity, tdTomato^+^ and tdTomato^−^ cells from mouse calvarial bone and cartilage were plated on fibrin gel (Millicell, Cat. MMA130, Millipore)-coated culture plates. Cells were plated at 3 × 10^5^ cells per well, and specific medium was added to each group. Tube formation was monitored for up to 12 hours, and a photographic record at 20x magnification was obtained.

### Statistics

All the data are presented as the means ± s.ds. The Shapiro–Wilk test for normality and Bartlett’s test for variance were performed to determine the appropriate statistical tests for the data analyses. Comparisons between two groups were assessed by Student’s t test, and comparisons among groups of samples were assessed by one-way ANOVA followed by Tukey’s multiple comparison test. The number of animals and the number of experimental repetitions are presented in the figure legends. GraphPad Prism (GraphPad Software, Inc., San Diego, CA, USA) was used for these analyses. (*P < 0.05, **P < 0.01, ***P < 0.000 1, NS = not statistically significant.)

## Supplementary information

Supplementary Figure 1

Supplementary Figure 2

Supplementary Figure 3

Supplementary Figure 4

Supplementary Figure 5

Supplementary Figure 6

Supplementary Figure 7

Supplementary Figure 8

Supplementary Figure 9

Supplementary Figure 10
