# Supplementary figures and images for: Type II collagen-positive progenitors are important stem cells in controlling skeletal development and vascular formation
Source: Bone Res. 2022 Jun 23;10:46. doi: 10.1038/s41413-022-00214-z (PMC9226163; doi:10.1038/s41413-022-00214-z)

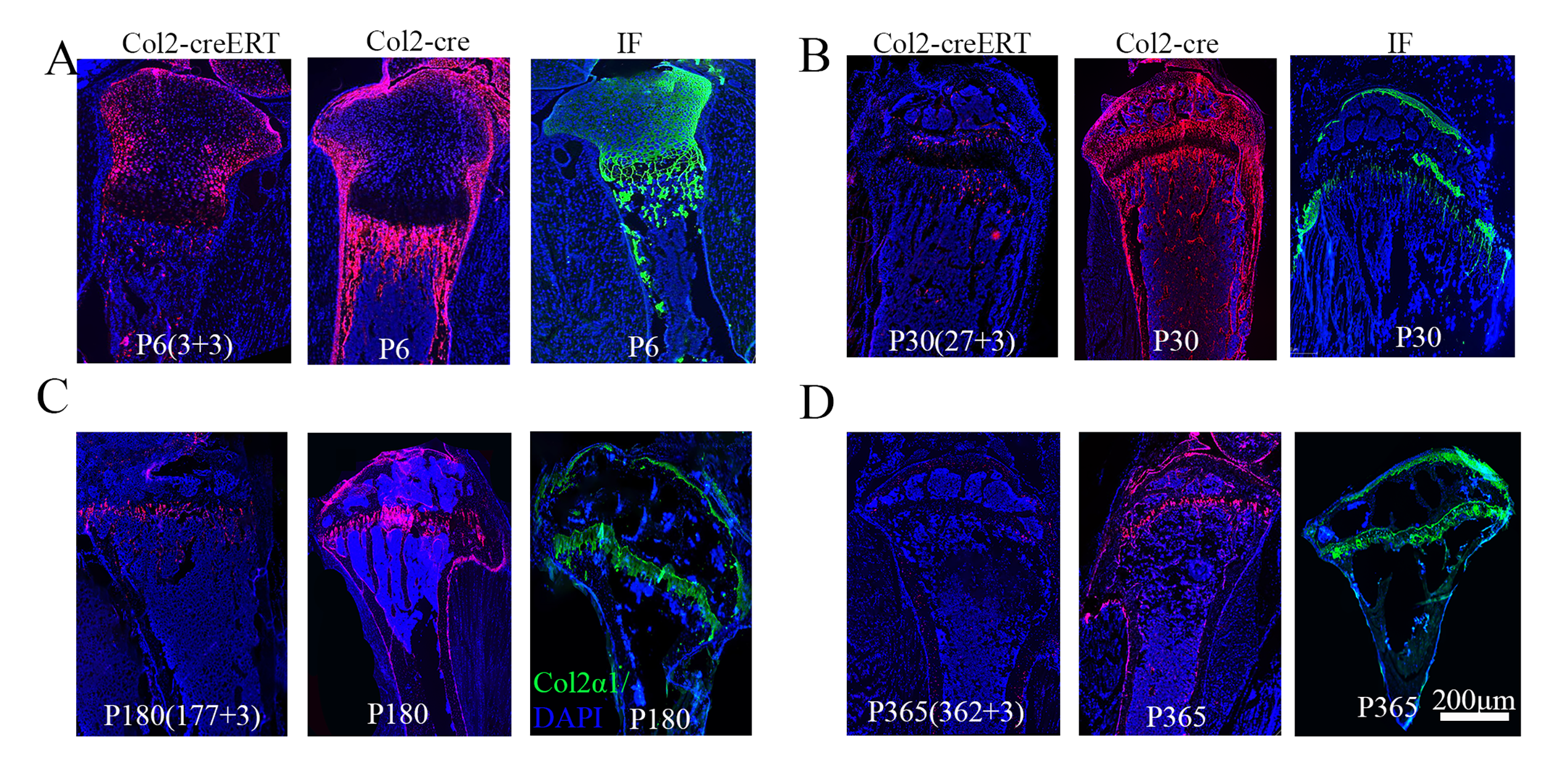

Supplement: Supplementary file 2 — Supplementary Figure 2 [file 41413_2022_214_MOESM2_ESM.tif]

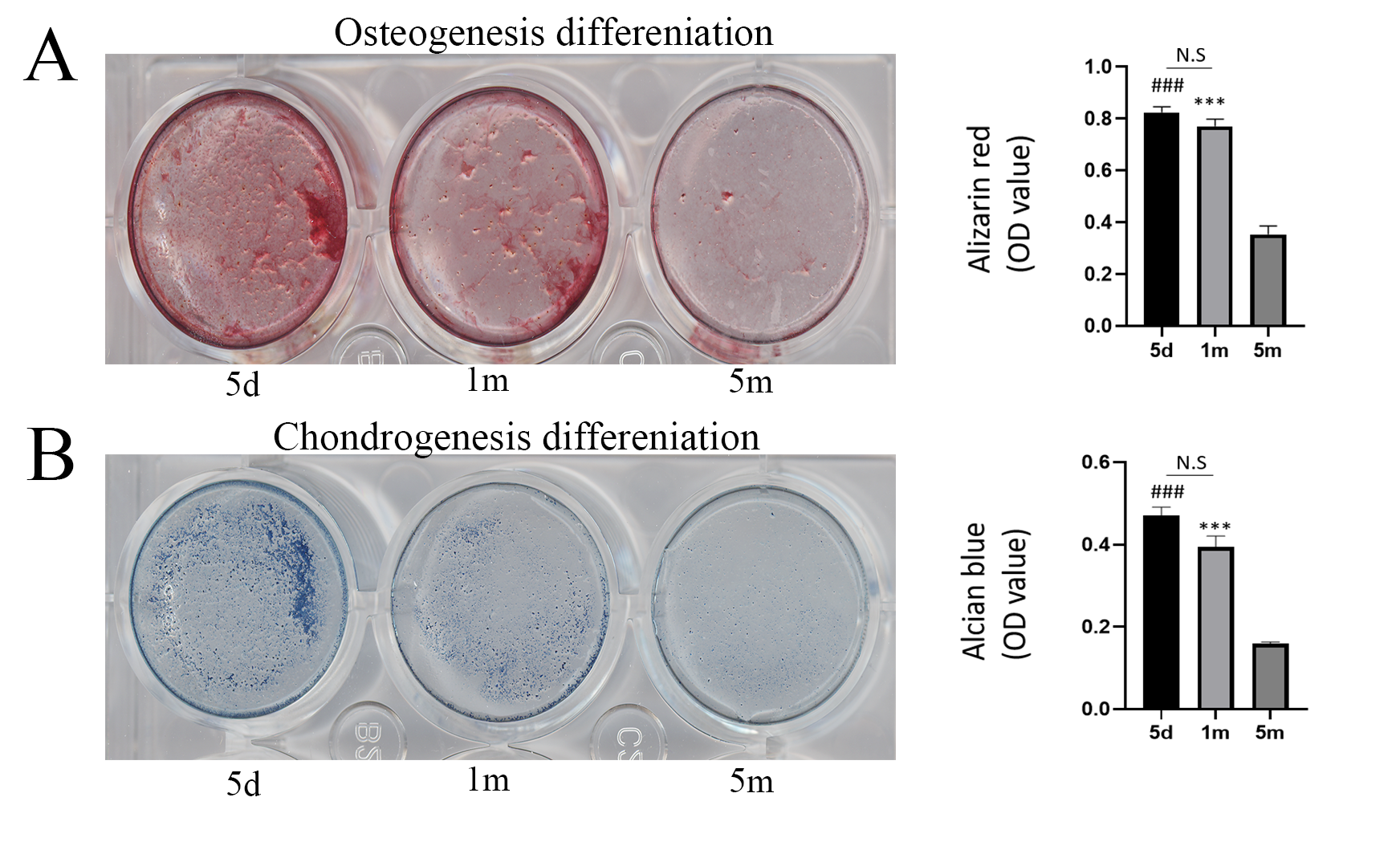

Supplement: Supplementary file 3 — Supplementary Figure 3 [file 41413_2022_214_MOESM3_ESM.tif]

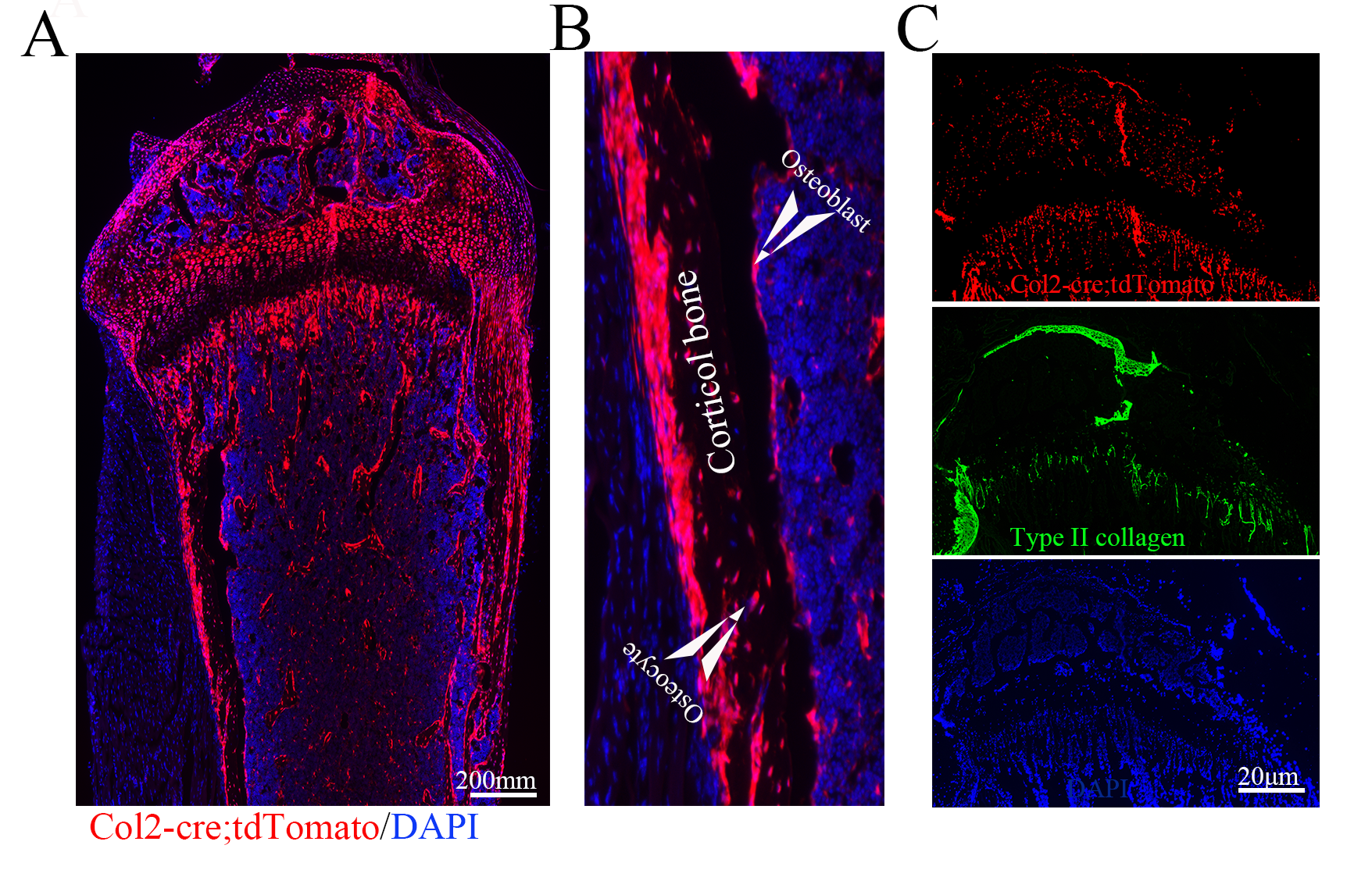

Supplement: Supplementary file 4 — Supplementary Figure 4 [file 41413_2022_214_MOESM4_ESM.tif]

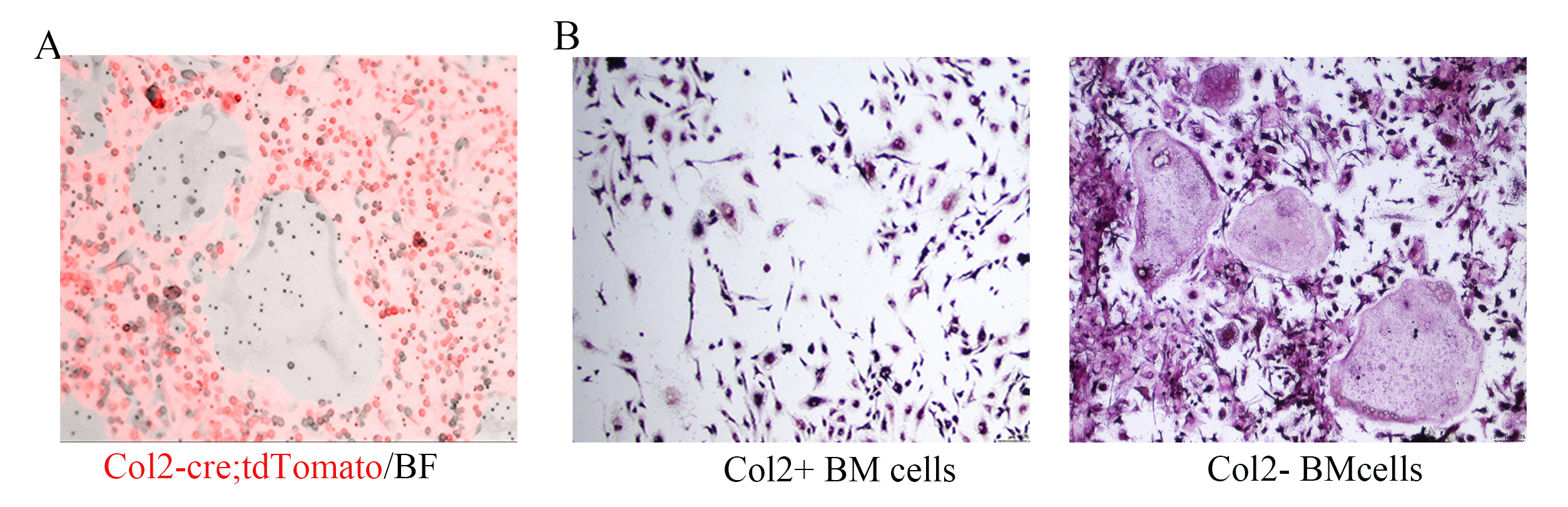

Supplement: Supplementary file 6 — Supplementary Figure 6 [file 41413_2022_214_MOESM6_ESM.tif]

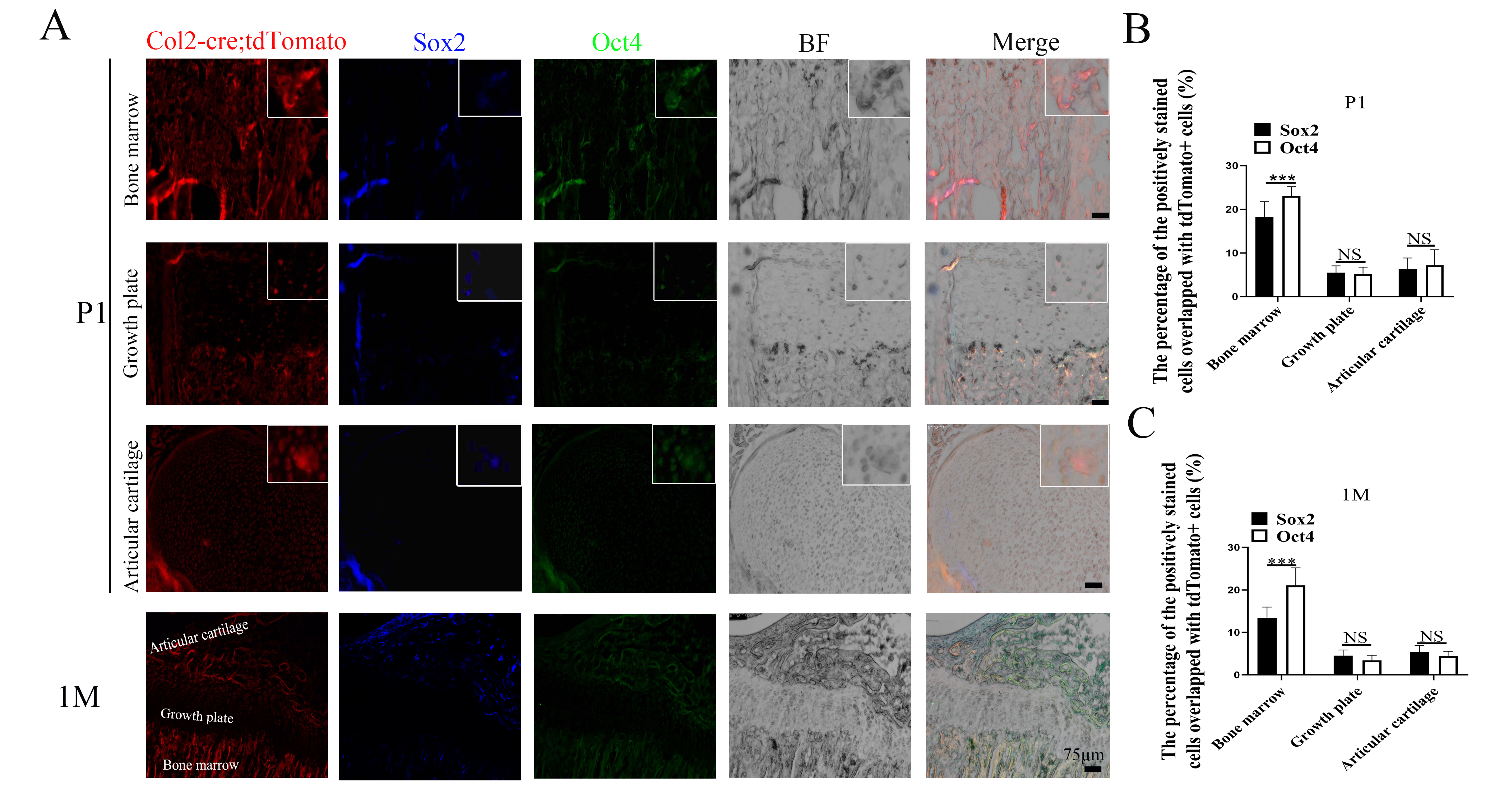

Supplement: Supplementary file 7 — Supplementary Figure 7 [file 41413_2022_214_MOESM7_ESM.tif]

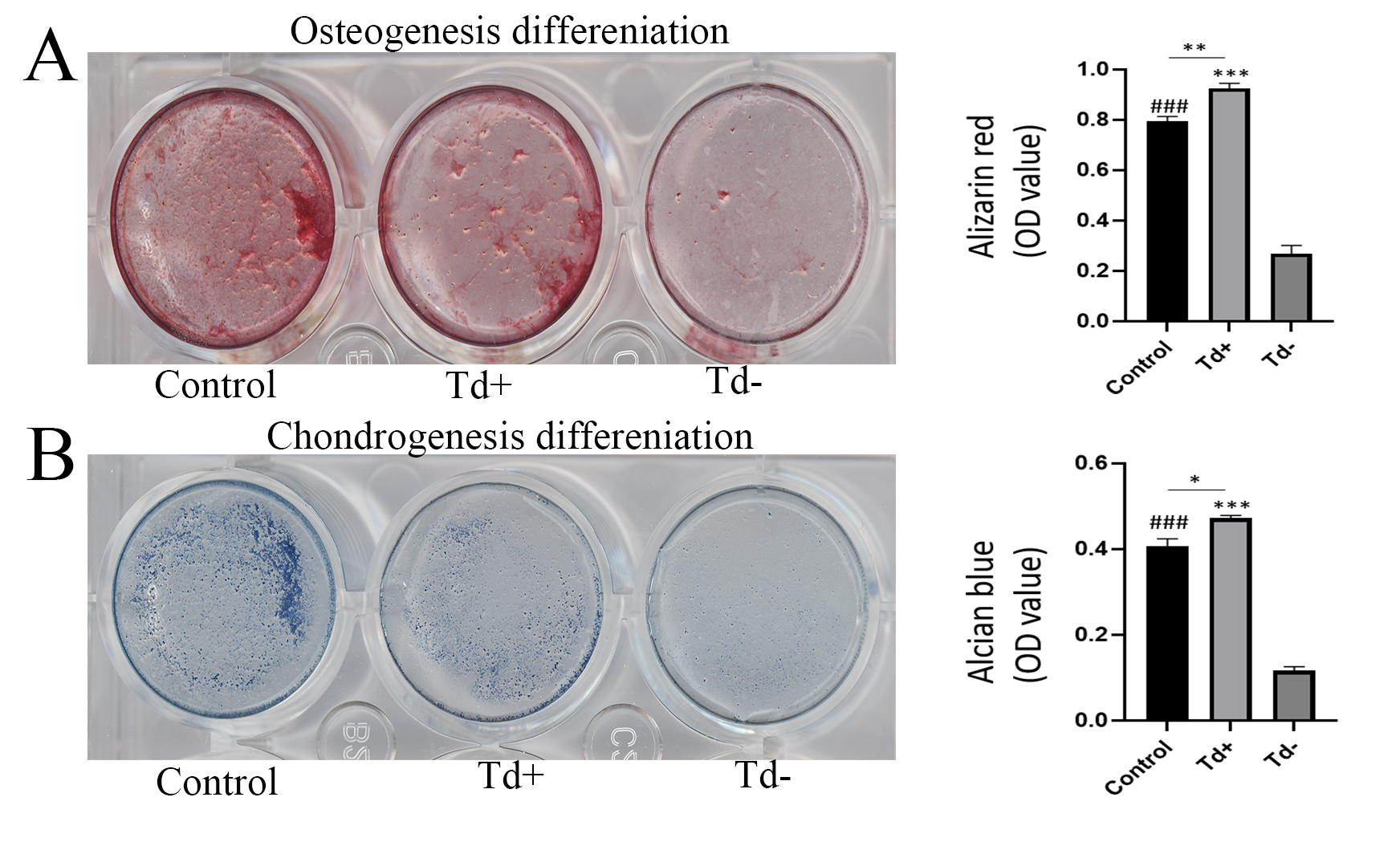

Supplement: Supplementary file 8 — Supplementary Figure 8 [file 41413_2022_214_MOESM8_ESM.tif]

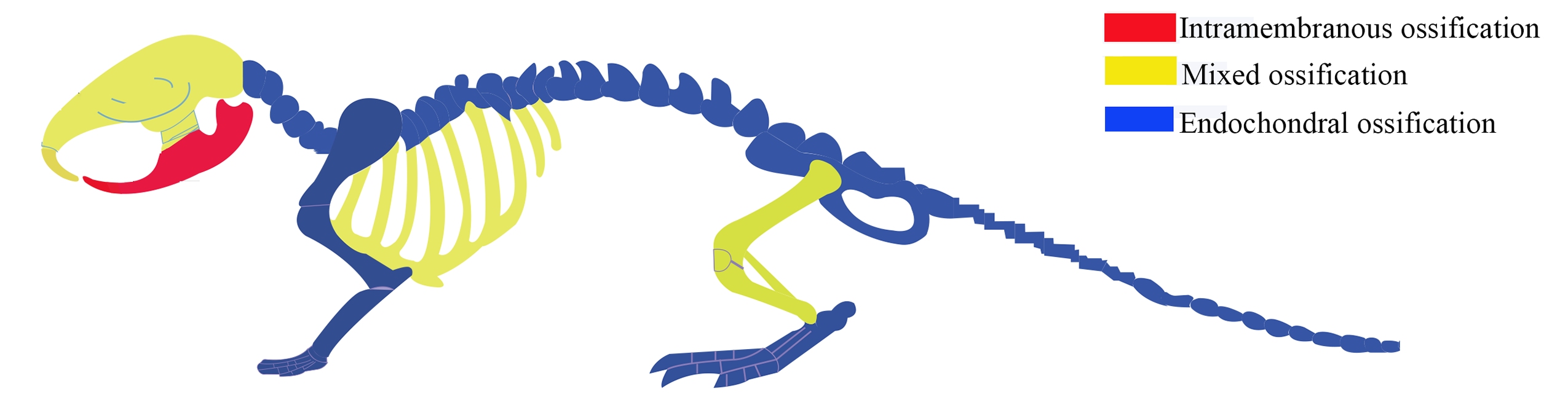

Supplement: Supplementary file 9 — Supplementary Figure 9 [file 41413_2022_214_MOESM9_ESM.tif]

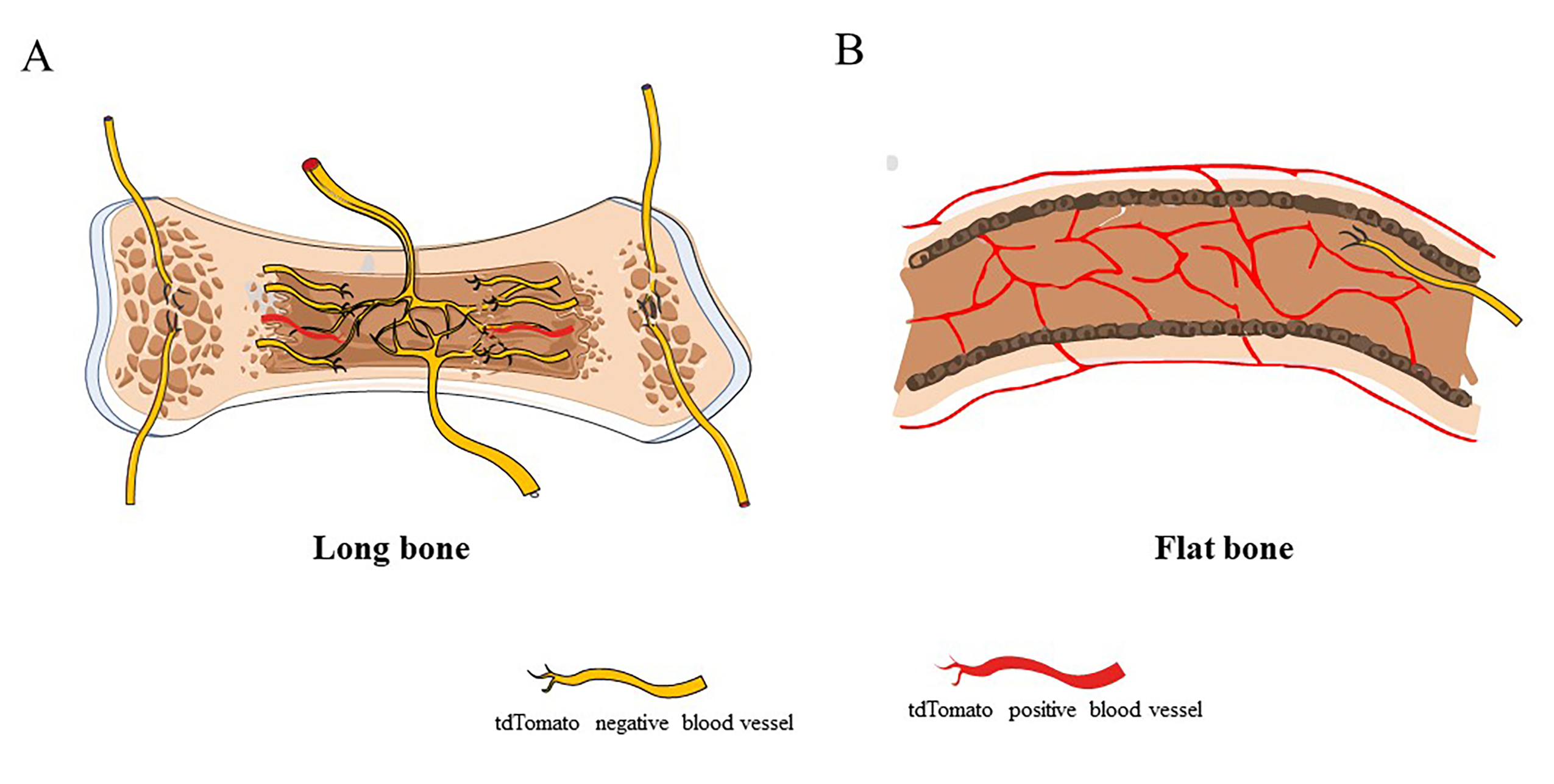

Supplement: Supplementary file 10 — Supplementary Figure 10 [file 41413_2022_214_MOESM10_ESM.tif]
